# From molecules to medicine: thiol selective bioconjugation in synthesis of diagnostic and therapeutic radiopharmaceuticals

**DOI:** 10.7150/thno.95469

**Published:** 2024-03-25

**Authors:** Iqra Bibi, Sajid Mushtaq, Kyo Chul Lee, Ji Ae Park, Jung Young Kim

**Affiliations:** 1 Affiliation Division of Applied RI, Korea Institute of Radiological & Medical Sciences (KIRAMS), 75 Nowon-ro, Nowon-gu, Seoul 01812, Republic of Korea.; 2 University of Science and Technology (UST), 217, Gajeong-ro, Yuseong-gu, Daejeon 34113, Republic of Korea.; 3 Department of Nuclear Engineering, Pakistan Institute of Engineering and Applied Sciences (PIEAS), P. O. Nilore, Islamabad 45650, Pakistan.

**Keywords:** Thiol-specific prosthetic groups, Radiopharmaceuticals, Imaging and radiotherapy, Maleimide

## Abstract

Radiolabeling of biomolecules and cells with radiolabeled prosthetic groups has significant implications for nuclear medicine, imaging, and radiotherapy. Achieving site-specific and controlled incorporation of radiolabeled prostheses under mild reaction conditions is crucial for minimizing the impact on the bioactivity of the radiolabeled compounds. The targeting of natural and abundant amino acids during radiolabeling of biomolecules often results in nonspecific and uncontrolled modifications. Cysteine is distinguished by its low natural abundance and unique nucleophilicity. It is therefore an optimal target for site-selective and site-specific radiolabeling of biomolecules under controlled parameters. This review extensively discusses thiol-specific radiolabeled prosthetic groups and provides a critical analysis and comprehensive study of the synthesis of these groups, their *in vitro* and *in vivo* stability profiles, reaction kinetics, stability of resulting adducts, and overall impact on the targeting ability of radiolabeled biomolecules. The insights presented here aim to facilitate the development of highly efficient radiopharmaceuticals, initially in preclinical settings and ultimately in clinical applications.

## Introduction

Advancements in modification of biomolecules such as proteins and antibodies have garnered considerable attention as a pivotal technology that is useful in deciphering* in vivo* and *in vitro* biochemical mechanisms and pioneering the development of innovative diagnostic and/or therapeutic radiopharmaceuticals 1-3. However, the synthesis of a variety of bioconjugates those are both structurally and functionally diverse using two or more small molecules and/or bio-macromolecules continues to be a challenge persisting in the fields of chemistry and biochemistry 4. Pivotal factors such as the specificity and site-selectivity of prosthetic groups and stability of conjugates need to be stringently controlled to safeguard the *in vivo* functionalities and inherent properties of biomolecules marked for modification. Hence, genetic engineering methodologies have been used adeptly to introduce specific chemical moieties at precise locations within the peptide or protein sequences. These strategically placed moieties act as anchoring points for subsequent radiolabeling via highly efficient and quantitative bioorthogonal chemical reaction 5. Alternatively, the direct modification of native antibodies and proteins has proven to be practical because it delves into the intrinsic reactivity profiles of various side-chain functionalities. Initial efforts to synthesize protein- or antibody-based radiopharmaceuticals involved the modification of relatively abundant, solvent-accessible, and highly reactive residues such as lysine 6. Therefore, lysine-specific prosthetic groups such as *N-*hydroxysuccinimide esters (NHS esters) or other groups that show excess availability were used for this purpose 7, 8. However, these approaches led to unpredictable modifications at multiple sites, resulting in heterogeneity and the potential blocking of biologically active sites 9. Currently, the availability of various prosthetic groups facilitates the radiolabeling of biomolecules with heightened specificity and efficiency 10. This advancement enables radiolabeling not only through lysine residues but also via other natural amino acids such as tyrosine, cysteine, and N-terminal cysteine 11.

**Figure 1:**
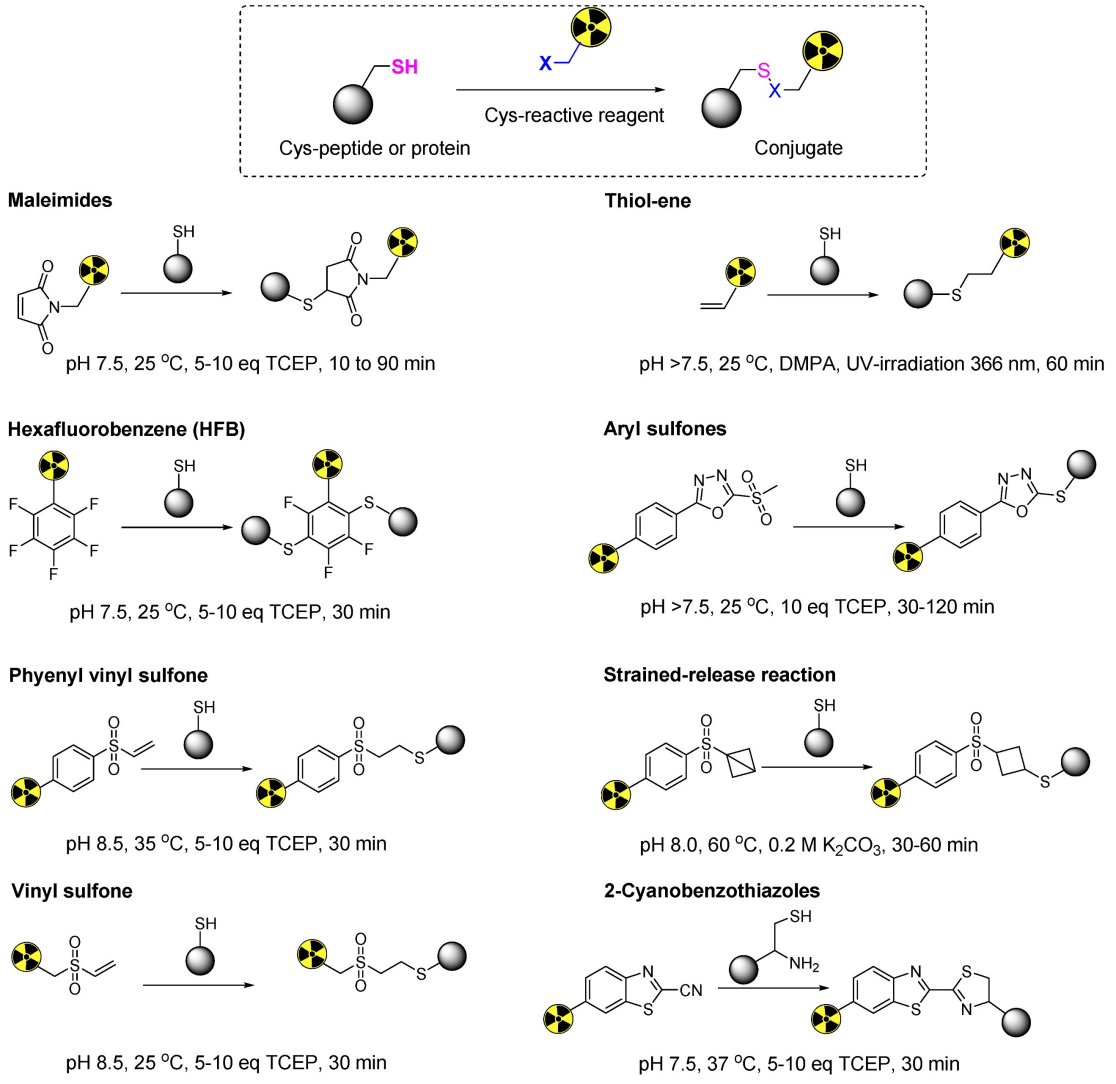
Representative thiol-specific prosthetic groups for the site-selective radiolabeling of cysteine containing biomolecules.

**Figure 2:**
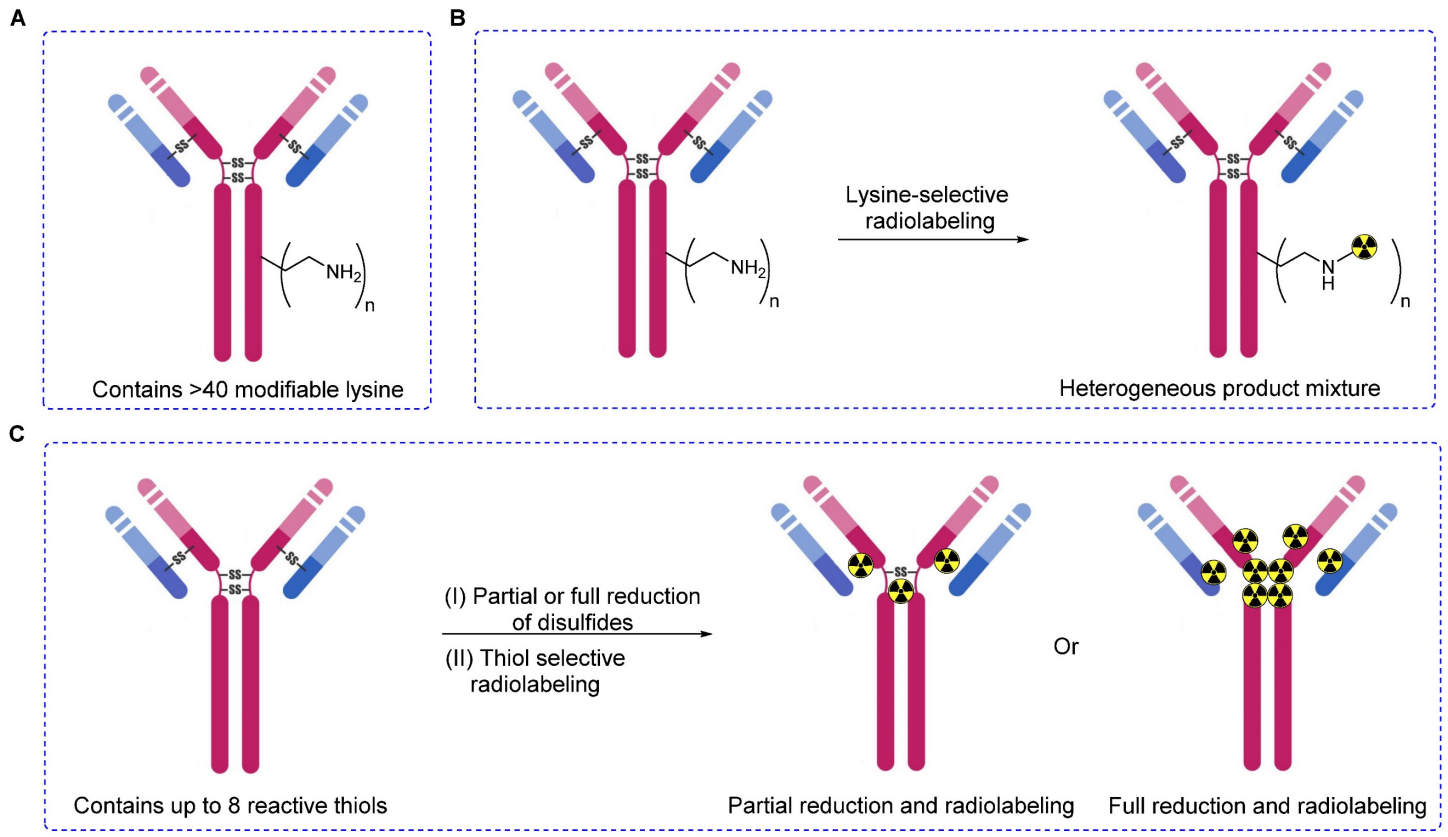
Lysine- or thiol-based radiolabeling of biomolecules. (A) Schematic diagram illustrating a monoclonal antibody with surface-exposed lysine residues and four disulfide bonds. (B) Non-specific and uncontrolled radiolabeling, as demonstrated by lysine-based methods, results in a heterogeneous mixture of products, potentially impacting the overall performance of radiopharmaceuticals. (C) Thiol-based radiolabeling of antibodies, following partial or complete reduction of disulfide bonds. Partial reduction, followed by radiolabeling, produces a heterogeneous mixture with a minimal degree of heterogeneity compared to lysine methods. Complete reduction, followed by a thorough radiolabeling reaction, yields a homogeneous product.

**Figure 3:**
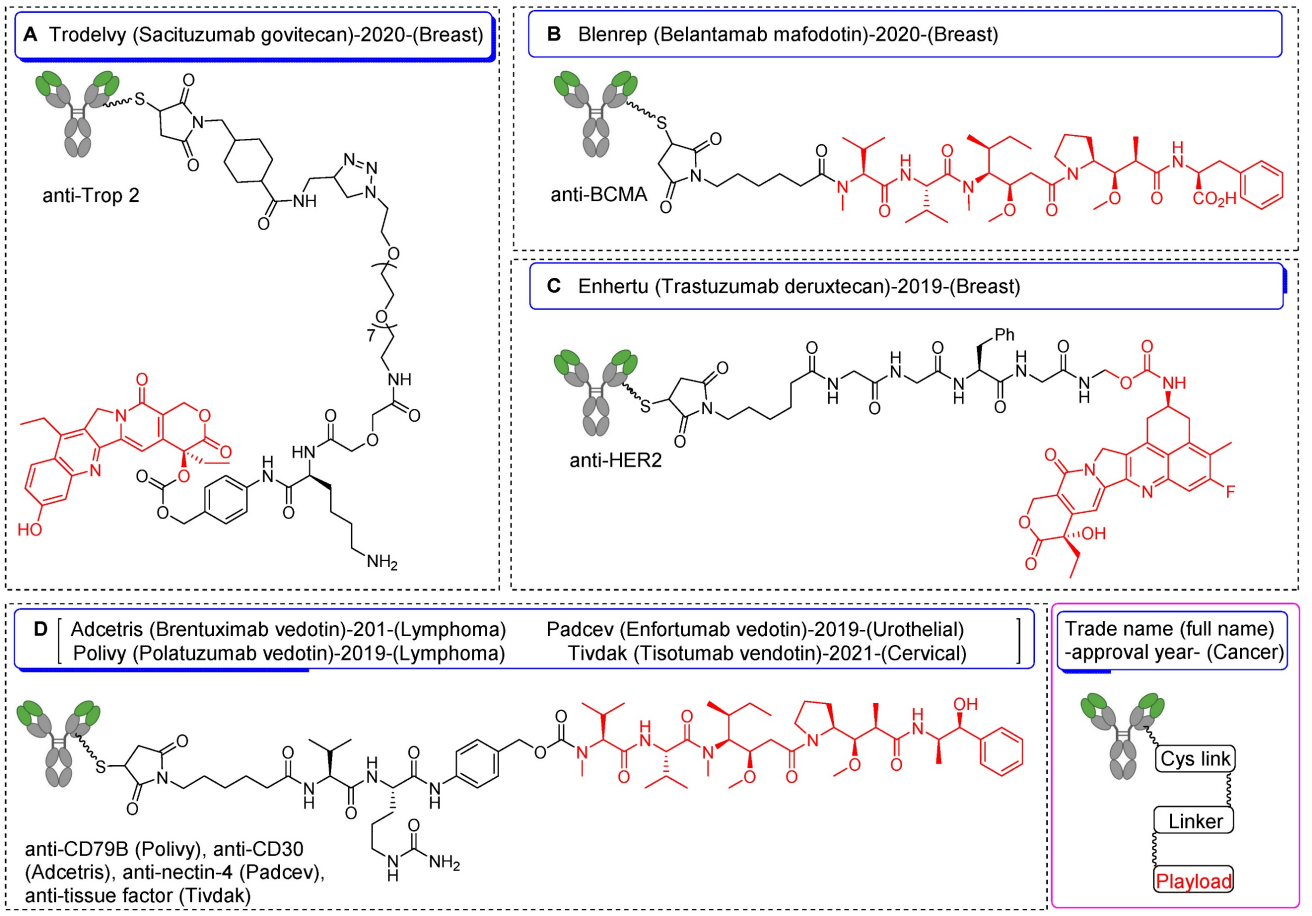
Schematic diagrams of FDA-approved antibody-drug conjugates synthesized utilizing Cys-based strategies. Information encompasses chemical structure of drug, trade name, full name, year of approval, targeted cancer type, and payload for each construct. (A) Sacituzumab govitecan, (B) belantamab mafodotin, (C) trastuzumab deruxtecan, and (D) brentuximab vedotin, enfortumab vedotin, polatuzumab vedotin, and tisotumab vedotin.

Among the spectrum of available amino acids, cysteine holds a distinct advantage as the most widely accepted amino acid for the precise and site-selective labeling of folded proteins and antibodies with a variety of prosthetic groups (Figure 1). Its prominence stems from its lower abundance compared with that of lysine and its unique nucleophilicity compared with its affinity to other reactive sites (Figure 2) 12. Moreover, the clinical acceptance of both developed and underdeveloped emerging therapeutic antibody-drug conjugates (ADCs) validates the superiority of cysteine-based modifications over other alternatives. This preference is rooted in the fact that monoclonal antibodies offer approximately eight cysteine and forty lysine residues suitable for prosthetic group conjugation. Consequently, it is anticipated that cysteine-based modifications yield a minimal level of heterogeneity compared to their lysine counterparts, thereby emphasizing their superior suitability for such applications 13. For example, several FDA-approved second-generation ADCs, including brentuximab vedotin, polatuzumab vedotin enfortumab vedotin, trastuzumab deruxtecan, sacituzumab govitecan, belantamab mafodotin, and tisotumab vedotin were predominantly modified via cysteine chemistry (Figure 3), demonstrating the effectiveness of this approach 14-16. Nevertheless, challenges persist in the form of conjugate instability, suboptimal reaction kinetics, and non-specific labeling reactions, which remain as primary concerns. These challenges have prompted innovation such as the design of novel and highly efficient prosthetic groups for the radiolabeling of cysteine-containing biomolecules. Although thiol-specific prosthetic groups have in general been reviewed extensively with regard to protein modifications 17-19, a comprehensive overview of the most recent advancements in thiol-specific prosthetic groups for radiolabeling micro- and macro-biomolecules is lacking. This review aims to fill the gap by discussing various facets, including the synthesis of radiolabeled thiol-specific prosthetic groups, their stability profiles, bioconjugation kinetics, and stability of the resulting adducts in relation to their chemical structures. Moreover, this review provides a critical analysis and comparative study aimed at aiding the scientific community in identifying the most effective methodologies for radiolabeling biomolecules. The insights presented here are intended to facilitate the development of superior radiopharmaceuticals, initially in preclinical settings and ultimately in clinical applications.

## Maleimide based radiolabeling

Michael acceptors, such as maleimide, represent an important class of prosthetic groups that react with cysteine to form thiosuccinimide bonds. Moore and Ward pioneered the initial exploration of the maleimide-thiol-based reaction and elucidated its potential for cross-linking thiol-containing proteins 20. Subsequently, maleimide-based prosthetic groups have been further developed for sulfhydryl group blocking, fluorescent mapping of cysteine residues in biomolecules, and the synthesis of therapeutic or diagnostic radiopharmaceuticals. Despite concerns regarding thiosuccinimide bond degradation, maleimide-thiol-based prostheses have gained significant traction and are currently well-established. Numerous studies have demonstrated their application in nuclear medicine. Currently, maleimide-based prosthetic groups are preferred by radiochemists for synthesizing radiopharmaceuticals using thiol-containing biomolecules. Several factors drive this preference. Maleimide-based reactions demonstrate high selectivity towards less-abundant thiol groups, yield minimal side products, and allow reactions to occur in aqueous media under mild conditions, and exhibit high reaction rates in the absence of heat and catalysts. The subsequent sections present a comprehensive examination of maleimide-based prosthetic groups.

### [^18^F]FPPD and [^18^F]DDPFB

**Figure 4:**
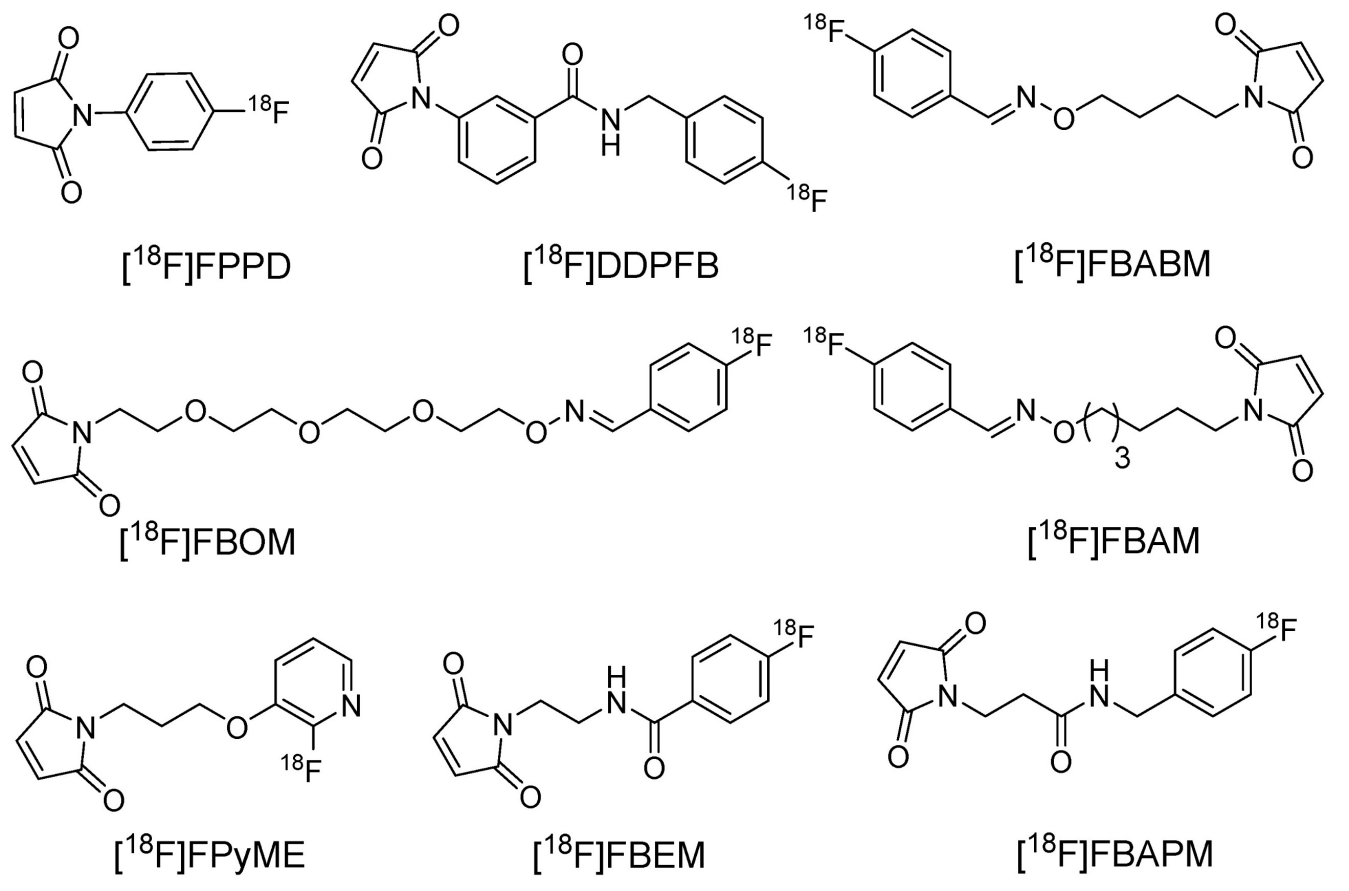
Representative** c**hemical structures of maleimide-based prosthetic groups for ^18^F-based radiolabeling of biomolecules.

In 1989, Hainfeld *et al.* conducted a pioneering study that investigated the use of maleimide for radiolabeling antibody fragments 21. Initially, the ^18^F radiolabeled prosthetic group N-(p-[^18^F] fluorophenyl)maleimide and its analogues were used in the pioneering application of labeling Fab structures. Notably, direct radiofluorination of the maleimide-based prosthetic group proved unfeasible due to the incompatibility of the base-labile maleimide with the essential basic conditions necessary for radiofluorination. Hence, an indirect approach was adopted through the synthesis of intermediate compounds such as p-[^18^F]Fluoroaniline and p-[^18^F]Fluorobenzonitrile to obtain the corresponding 1-(4-[^18^F]fluorophenyl)-pyrrole-2,5-dione ([^18^F]FPPD) and *N*-[3-(2,5-dioxo-2,5-dihydropyrrol-1-yl)-phenyl]-4-[^18^F]fluorobenzamide ([^18^F]DDPFB) compounds, respectively (Figure 4). Although both prosthetic groups were radiolabeled with high yields, only [^18^F]DDPFB was investigated further. [^18^F]DDPFB successfully radiolabeled rabbit IgG-derived Fab with >50% radioactivity attached to the antibody fragment. However, this study did not assess the *in vitro* stability or perform *in vivo* testing using the radiolabeled antibody.

### [^18^F]FBABM, [^18^F]FBAM, and [^18^F]FBOM

**Scheme 1:**
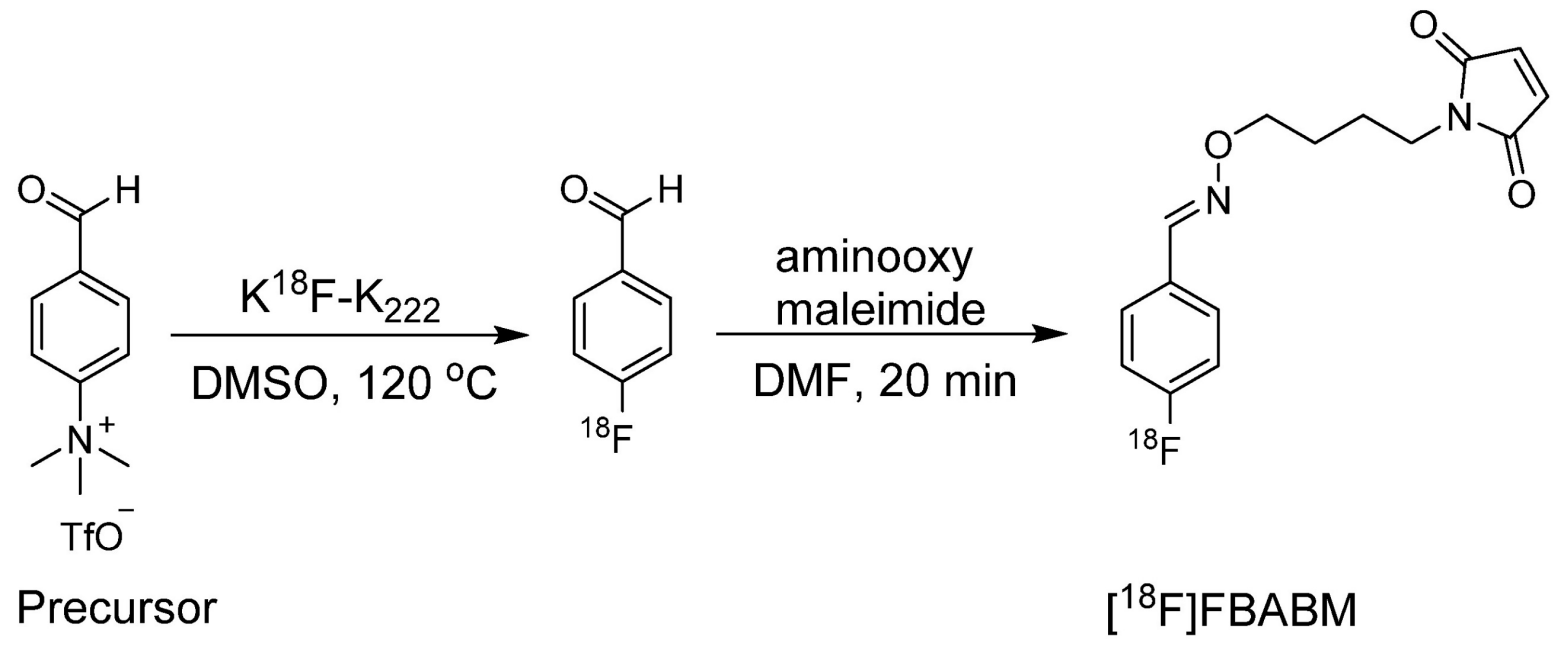
Synthesis of [^18^F]FBABM utilizing reaction between [^18^F]fluorobenzaldehyde and aminooxy maleimide.

During the synthesis of [^18^F]DDPFB, utilizing m-maleimidobenzoyl-*N*-hydroxysuccinimide ester (MBS) following ^18^F radiolabeling was considered inefficient because of the poor stability of the NHS ester. An alternative approach involved the synthesis of ^18^F labeled maleimide-based prosthetic group through the reaction of benzaldehyde with aminooxy group. The selection of the aminooxy functional group over the amine group occurs because of its high reactivity and selectivity towards the carbonyl group. Moreover, oxime ethers exhibit exceptional stability, which is crucial for efficient radiolabeling. In this study, 4-[^18^F]fluorobenzaldehyde was synthesized by the radiofluorination of 4-(formylphenyl)-trimethylammonium triflate at 120 °C for 10 min. Subsequently, the purified 4-[^18^F]fluorobenzaldehyde was treated with aminooxy maleimide to yield the corresponding [^18^F]-labeled maleimide (*N*-[4-[(4-[^18^F]fluorobenzylidene)aminooxy]butyl]maleimide ([^18^F]FBABM) (Scheme 1) at a moderate radiochemical yield (>35%).^18^F labeled maleimide was further used for radiolabeling the linear peptide Glutathione (GSH) and thiol containing antisense oligodeoxynucleotides (ODNs). Radiolabeled GSH and ODNs were obtained at moderate yields of 70% and 5%, respectively. However, the radiolabeled compounds were not further analyzed in biological studies 22. Wuest *et al*. used a similar strategy to synthesize N-[6-(4-[^18^F]fluoro-benzylidene) aminooxyhexyl]maleimide ([^18^F]FBAM) with 29% total radiochemical yield. This prosthetic group was used to radiolabel various small peptides such as GSH and human low-density lipoprotein (LDL). ^18^F-labeled LDL (17 ± 10% radiochemical yield) demonstrated high *in vitro* and *in vivo* stabilities 23. To enhance the hydrophilicity of [^18^F]FBAM, some researchers tried to synthesize 4-[^18^F]Fluorobenzaldehyde-O-(2-{2-[2-(pyrrol-2,5-dione-1-yl)ethoxy]ethoxy}- ethyl)oxime ([^18^F]FBOM). However, the radiochemical yield of [^18^F]FBOM and its radiolabeling efficiency for LDL protein were suboptimal compared with those of [^18^F]FBAM 24.

### [^18^F]FPyME

To synthesize radio-fluoropyridinyl moieties, nucleophilic heteroaromatic substitution with [^18^F]fluoride has emerged as a more precise strategy for synthesizing tracers with high specific radioactivity. Based on this, Bruin *et al.* detailed the synthesis of [^18^F]fluoropyridine-based maleimide for the highly specific radiolabeling of biomolecules 25. They synthesized [(2-nitro-pyridin-3-yloxy)alkyl]carbamic acid *tert*-butyl esters for radiofluorination and concurrently investigated the influence of alkyl chain length on the radiolabeling reaction. The nitro moiety acts as an efficient leaving group without requiring additional electron-withdrawing groups to activate the heteroaromatic ring. Precursor [(2-nitro-pyridin-3-yloxy)alkyl]carbamic acid *tert*-butyl ester (alkyl: ethyl, propyl, butyl, pentyl or hexyl) was heated in the presence of dried K[^18^F]F-K_222_ complex at 145 °C for 30 min to obtain the corresponding ^18^F-labeled compound. Notably, the precursor with a propyl chain exhibited a high radiochemical yield (50-75%) and was chosen for subsequent investigation. Incorporation of the maleimide group into the radiofluorinated compound was achieved through the reaction of maleic anhydride or *N*-methoxycarbonylmaleimide in o-xylene under reflux conditions. However, *N*-methoxycarbonylmaleimide provided a superior radiochemical yield (46-77%) for the synthesis of 1-[3-(2-[^18^F]Fluoropyridin-3-yloxy)propyl]pyrrole-2,5-dione ([^18^F]FPyME) (Figure 4). [^18^F]FPyME was also synthesized using ([3-(3-*tert*-butoxycarbonylaminopropoxy)-pyridin-2-yl]trimethylammonium trifluoromethane sulfonate) as an alternative to the nitro-based leaving group, resulting in a high radiochemical yield under relatively mild reaction conditions. The efficacy of [^18^F]FPyME was assessed using (*N*-Ac)-KAAAAC as the model peptide. Radiolabeling was achieved with high yields (60-70%, non-decay-corrected); however, the radiolabeled compounds were not subjected to further biological studies.

### [^18^F]FBEM

Despite numerous reports of maleimide-based radiolabeled compounds, comprehensive *in vivo* biodistribution studies have been notably lacking. Addressing this gape, Cai *et al*. synthesized N-[2-(4-[^18^F]fluorobenzamido)ethyl]maleimide ([^18^F]FBEM) as a novel maleimide-based prosthetic group for *in vivo* investigations 26. The synthesis of [^18^F]FBEM involves the coupling of N-(2-aminoethyl)maleimide with N-succinimidyl-4-[^18^F]fluorobenzoate ([^18^F]SFB) under basic conditions, resulting in a modest radiochemical yield (5%). Subsequently, [^18^F]FBEM was used to labeled thiol-containing c(RGDyK) peptide in both monomeric (SRGD) and dimeric (SRGD2) forms, providing high radiochemical yields of [^18^F]FBEM-SRGD and [^18^F]FBEM-SRGD2 (≥85 ± 5% non-decay corrected) at >98% purity. Cell-binding affinity studies of FBEM-SRGD and FBEM-SRGD2 were conducted in U87MG cells and compared with those of c(RGDyK) and c(RGDyK)2. The IC50 values for FBEM-SRGD (66.8 ± 5.1 nmol/L), FBEM-SRGD2 (55.1 ± 6.5 nmol/L), c(RGDyK) (51.3 ± 4.2 nmol/L), and c(RGDyK)2 (26.1 ± 3.2 nmol/L) were comparable, suggesting that maleimide-based conjugation exerted no discernible impact on the integrin binding affinity of the peptides. [^18^F]FBEM-SRGD2 demonstrated enhanced *in vitro* and *in vivo* stability for up to 6 h, exhibiting high α_v_β_3_ integrin affinity *in vitro* and *in vivo*. Its rapid renal clearance and favorable pharmacokinetics further enhance the tumor-to-background contrast and imaging quality. In another study, Chen *et al*. proposed ^18^F radiolabeling of a novel GLP-1 analog (EM3106B) using the [^18^F]FBEM prosthetic group. GLP-1 receptors play a pivotal role as crucial targets not only in GLP-1R expressed pancreatic beta cells but also in insulinoma (INS) 27, 28. [^18^F]FBEM-EM3106B was highly stable in human plasma and mouse serum. It exhibited high binding affinity (IC_50_ 1.07 ± 0.48 nM) toward GLP-1R positive insulinomas cells, comparable to that of EM3106B (IC_50_ 1.25 ± 0.37 nM) alone. PET imaging data revealed elevated uptake of radiotracer in GLP-1R positive insulinomas compared with that in low GLP-1R expressed MDA-MB-435 tumors. The specificity of [^18^F]FBEM-EM3106B towards tumors was confirmed using blocking assays 29. They additionally explored the radiolabeling of exendin-4, [Cys^0^] and [Cys^40^] using [^18^F]FBEM. Both radiotracers ([^18^F]FBEM-[Cys^40^]-exendin-4 and [^18^F]FBEM-[Cys^0^]-exendin-4) exhibited high binding affinities for GLP-1R-positive insulinomas cells. However, [^18^F]FBEM-[Cys^40^]-exendin-4 demonstrated superior uptake in INS xenograft models, demonstrating its potential as a radiotracer for further studies 30. In another example, an [^18^F]FBEM analog 4-[^18^F]fluorobenzylamidopropionyl maleimide ([^18^F]FBAPM) was synthesized through a series of four radiolabeling steps, yielding a decay-corrected output of 55% in 70 min with a specific activity range of 50-70 GBq/μmol. The radiolabeling efficiency of [^18^F]FBAPM was assessed using the GSH peptide 31.

### [^18^F]FDG-HMO

**Scheme 2:**
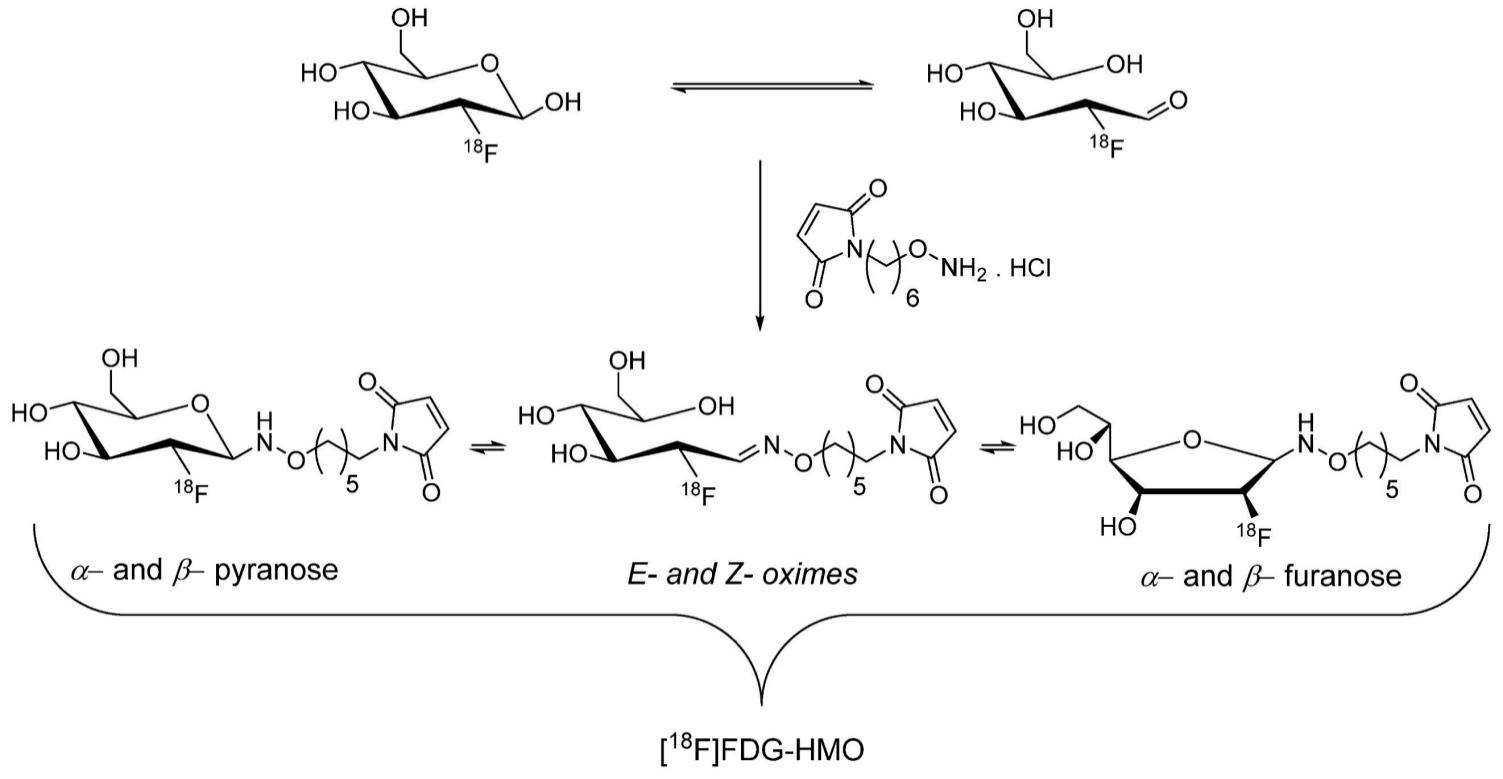
The proposed aminooxy-aldehyde coupling reaction between aminooxy maleimide hydrochloride with [^18^F]FDG for the synthesis of [^18^F]FDG-HMO. [^18^F]FDG-HMO exists in the form of isomers.

[^18^F]Fluoro-2-deoxy-D-glucose ([^18^F]FDG) is a cornerstone radiopharmaceutical for clinical PET imaging because of its exceptional efficiency and ease of automated and streamlined radiosynthesis process, which enables its widespread availability in cancer hospitals. Despite this prevalence, the incorporation of [^18^F]FDG as a building block or prosthetic group in radiopharmaceutical synthesis remains relatively limited. In 2008, Wuest *et al*. proposed that the abundant accessibility of [^18^F]FDG in hospitals could be harnessed to synthesize maleimide-based prosthetic groups. Notably, [^18^F]FDG exists as a mixture of α/β anomers in aqueous solutions with an accompanying transformation into an acyclic aldehyde form through a ring opening mechanism. They further aimed to synthesize [^18^F]FDG-maleimide prosthetic groups through a chemoselective aminooxy-aldehyde coupling reaction in anticipation of the utility of the aldehyde form. Treatment of aminooxy maleimide hydrochloride with [^18^F]FDG at 100 °C for 15 min produced an isomeric mixture of [^18^F]FDG-MHO (Scheme 2) with high radiochemical yield (45-69%) and purity (>95%), which was purified using HPLC. The efficiency of [^18^F]FDG-MHO was demonstrated by its successful conjugation with the model peptide glutathione (GSH) and protein annexin A5. Notably, incubation of [^18^F]FDG-MHO with annexin A5 yielded the corresponding ^18^F-labeled protein with high radiochemical yield (43-58%) and purity. Its subsequent application in PET imaging of human colorectal adenocarcinoma (HT-29) animal model revealed excellent *in vivo* stability of the [^18^F]FDG-MHO-anxA5 complex. Although a significant radioactivity signal was observed in the kidneys and liver, tumor uptake remained relatively limited in these experimental settings 32.

### Radiometal chelator based maleimide prosthetic groups

**Figure 5:**
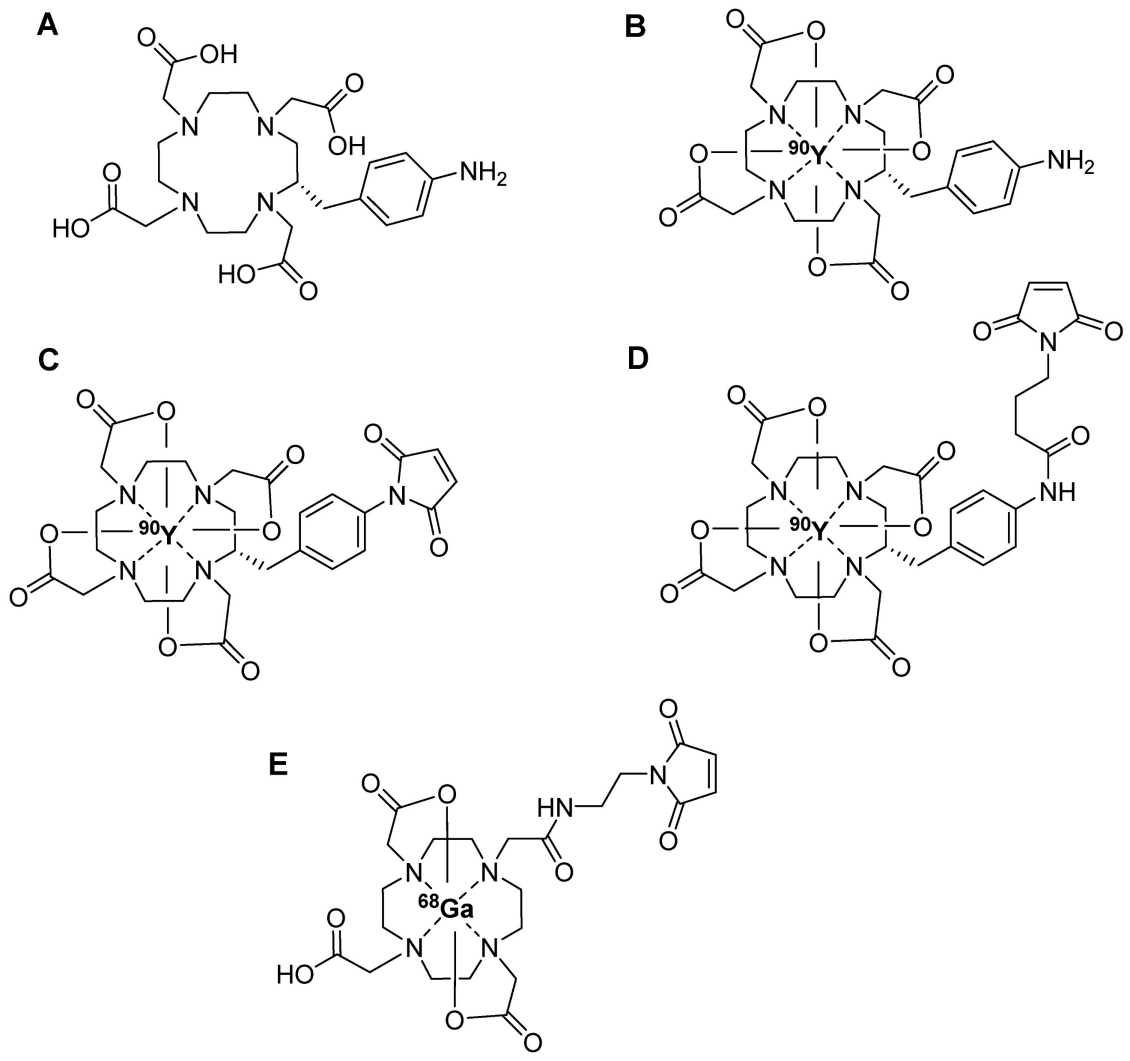
Chemical structures of radiometal chelator based maleimide prosthetic groups. (A) DOTA-NH_2,_ (B) ^90^Y-labeled DOTA-NH_2_, (C)^ 90^Y-DOTA-Mal-1, (D)^ 90^Y-DOTA-Mal-2, and (E) ^68^Ga-labeled DOTA-Mal.

Trivalent radionuclides, in particular, are combined with biologically active molecules using bifunctional chelators (BFCs) such as (1,4,7,10-tetraazacyclododecane-1,4,7,10-tetrayl)-tetraacetic acid (DOTA) 33. DOTA is renowned for its remarkable capability to form highly stable complexes with theranostic radioisotopes like lutetium-177 (^177^Lu) and yttrium-90 (^90^Y), exhibiting superior kinetic inertness *in vivo* 34, 35. However, the relatively sluggish rate of complexation and low radiolabeling yield associated with DOTA chelator necessitate reactions at elevated temperature (>80 °C), limiting its use in radiolabeling sensitive biomolecules. The alternative direct radiolabeling approach involves the use of pre-radiolabeled prosthetic groups. Steinbach *et al*. introduced a maleimide-derived DOTA prosthetic group labeled with yttrium-90 (^90^Y), designed for the targeted radiolabeling of biomolecules containing thiol groups in a site-selective manner. They radiolabeled [(2S)-2-(4-Aminobenzyl)-1,4,7,10-tetraazacyclododecane-1,4,7,10-tetrayl]-tetraacetato (DOTA-NH_2_) (Figure 5A) with ^90^Y at 95 °C for 20 min (Figure 5B) and subsequently treated it with *N*-methoxycarbonylmaleimide or *N*-(*γ*-Maleimidobutyryloxy)succinimide ester under basic conditions at room temperature. The reaction yielded the corresponding *N*-aryl maleimide (^90^Y-DOTA-Mal-1, Figure 5C) and the *N*-alkyl maleimide (^90^Y-DOTA-Mal-2, Figure 5D), respectively. Of these, ^90^Y-DOTA-Mal-2 exhibited superior hydrolytic stability (>96 h, pH = 8.0) compared with that of ^90^Y-DOTA-Mal-1. Owing to its heightened stability, ^90^Y-DOTA-Mal-2 was used in the radiolabeled model peptide GSH and the model macromolecule 12mer L-RNA. Both biomolecules were radiolabeled with a high radiochemical yield; however, direct radiolabeling of DOTA-L-RNA provided a better overall radiochemical yield 36. Similarly, Cheng *et al*. used ^68^Ga radiolabeled maleimido-mono-amide-DOTA (^68^Ga-DOTA-Mal, Figure 5E) for ^68^Ga radiolabeling of bovine serum albumin (^68^Ga-DOTA-BSA), thiol containing cyclic RGD (^68^Ga-DOTA-RGD), and folic acid (^68^Ga-DOTA-FA). The radiolabeling yields of ^68^Ga-DOTA-BSA**,** ^68^Ga-DOTA-FA, and ^68^Ga-DOTA-RGD were 20%, 85%, and 90%, respectively. PET imaging and biodistribution data were gathered for ^68^Ga-DOTA-BSA, ^68^Ga-DOTA-FA, and ^68^Ga-DOTA-RGD in mice with inflammation, SKOV-3 tumors, and MGC-803 tumors, respectively. PET imaging revealed significant uptake of radiolabeled compounds in the corresponding tumors or inflammatory areas in all animal models 37. The radiolabeled prosthetic group did not impact the binding affinity and pharmacokinetics of the radiolabeled biomolecule.

**Figure 6:**
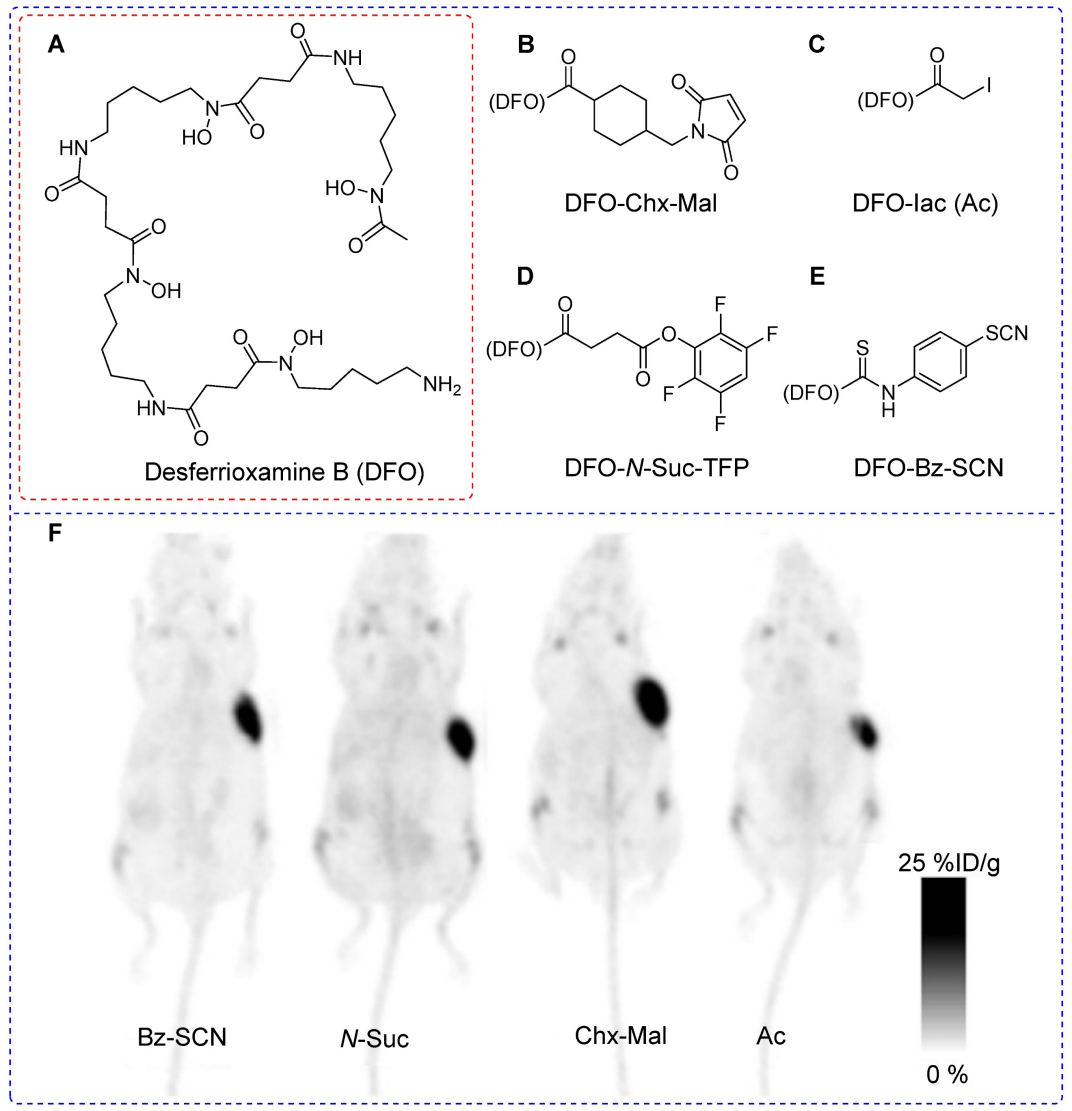
Desferrioxamine B (DFO) based prosthetic groups for thiol- or amino- based ^89^Zr radiolabeling of trastuzumab antibody and pharmacokinetic study using PET imaging. Schematic structure of (A) Desferrioxamine B (DFO), (B) DFO-CHx-Mal, (C) DFO-Iac, (D) DFO-*N-*Suc-TFP, and (E) DFO-Bz-SCN. (F) PET imaging data were acquired for all four ^89^Zr-trastuzumab derivatives using BT474M1 xenograft models (96 h post intravenous injection). Adapted with permission from 43, copyright 2010, Elsevier Inc.

Successful immuno PET imaging relies on the selection of radioisotopes with half-lives that are compatible with those of the biomolecules. For instance, antibodies (approximately 140 kDa) have a biological half-life ranging from several days to weeks; yet an optimal tumor-to-background ratio typically manifests 2-6 days after administration 38. In this context, positron-emitting radioisotopes such as zirconium-89 (^89^Zr, T_1/2_ = 3.3 days) represent a better solution compared with ^18^F or ^68^Ga 39. ^89^Zr has been integrated into biomolecules using lysine 40 and cysteine 41. Monoclonal antibodies offer approximately 8 cysteine and 40 lysine sites for prosthetic group conjugation. Predictably, cysteine-based modifications aim to reduce heterogeneity compared with lysine-based modifications. However, cysteine-based modifications may decrease the plasma half-lives of the diagnostic or therapeutic drugs. Tinianow *et al.* evaluated the impact of thiol- or amino-based radiolabeling on the bioactivity of a radiopharmaceutical by conducting a comparative study using the desferrioxamine B (DFO) chelator (Figure 6A) containing thiol-based prosthetic groups such as DFO-Chx-Mal (Figure 6B) 42 and DFO-Iac (Figure 6C) along with frequently reported lysine-based prosthetic groups such as DFO-*N*-Suc-TFP (Figure 6D) and DFO-Bz-SCN (Figure 6E). All four prosthetic groups were used to incorporate DFO chelator into trastuzumab under the given reaction conditions. Notably, DFO-Chx-Mal demonstrated superior conjugation under mild conditions than did DFO-Iac. Moreover, DFO-Chx-Mal exhibited greater radiochemical yield and purity than that of DFO-Iac. The *in vitro* stability of DFO-Chx-Mal matched or surpassed that of DFO-*N*-Suc-TFP, DFO-Bz-SCN, and DFO-Iac for up to 5 days in mouse serum under physiological conditions; although a gradual loss of antibody-bound ^89^Zr (1.8% per day) was observed. PET imaging and biodistribution data were acquired for all four ^89^Zr-trastuzumab derivatives using the BT474M1 xenograft model (Figure 6F). Despite slight differences in tumor, kidney, bone, and muscle uptake, all prosthetic groups exhibited comparable biodistribution and imaging outcomes. However, using site-selective DFO-Chx-Mal offers distinct advantages. Notably, site-sensitive radiolabeling of genetically modified antibodies avoids potential alterations in antigen binding sites and ensures homogeneous and nearly identical products with minimal batch-to-batch variability 43.

**Figure 7:**
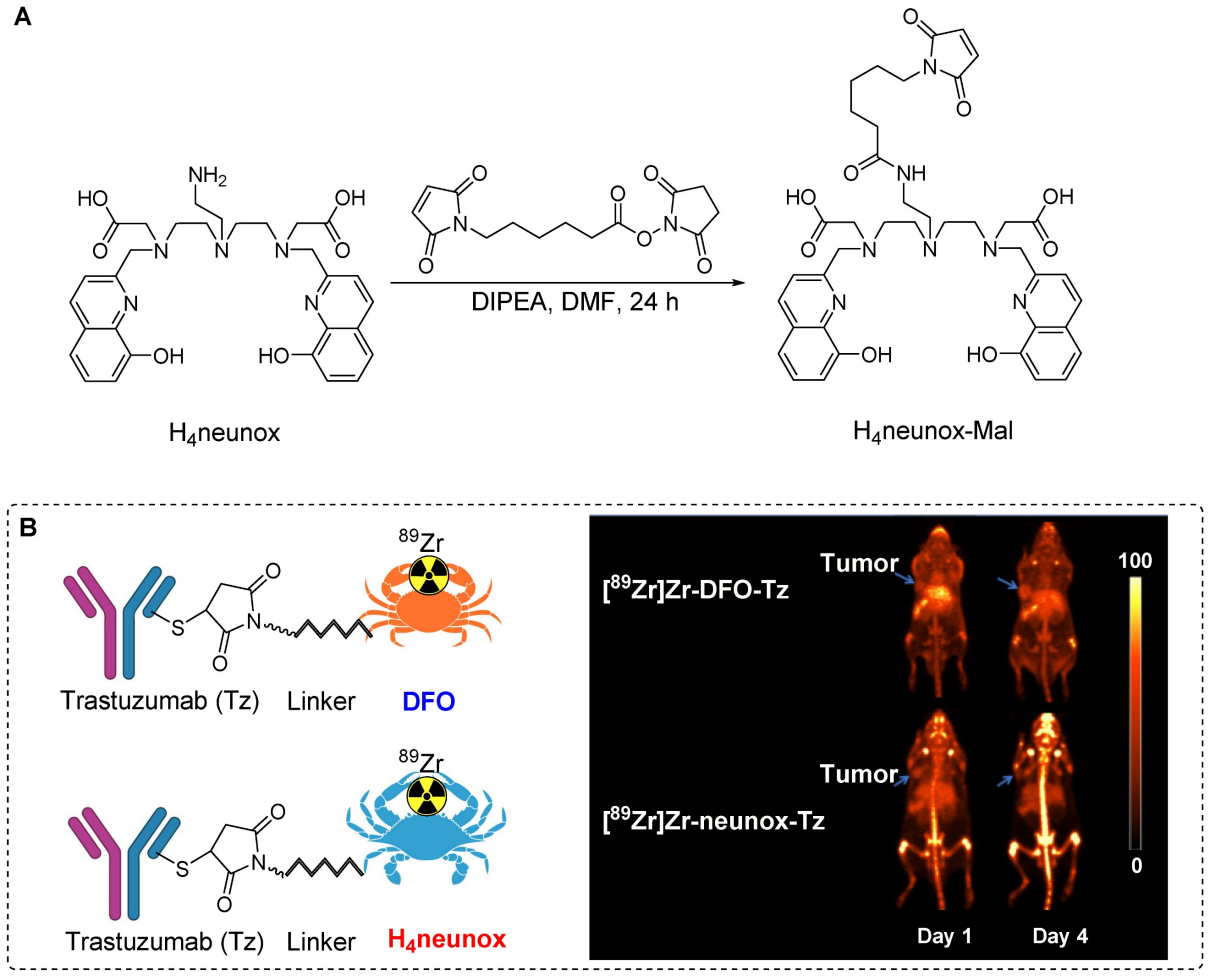
Illustration depicting the synthesis process of H_4_neunox-Mal and its *in vivo* studies. (A) The synthesis of H_4_neunox-Mal involves the use of *N*-Succinimidyl 6-maleimidohexanoate and H_4_neunox under basic conditions. (B) *In vivo* PET imaging data of [^89^Zr]Zr-DFO-trastuzumab and [^89^Zr]Zr-neunox-trastuzumab, conducted in a SKOV-3 cancer xenograft model on one and four days post-intravenous injection. Adapted with permission from 44, copyright 2022, Elsevier Inc.

In pursuit of a chelating agent with efficiency that surpasses that of DFO, Orvig *et al*. devised the synthesis of 2,2'-((((2-aminoethyl)azanediyl)bis(ethane-2,1-diyl))bis(((8-hydroxyquinolin-2-yl)methyl)azanediyl))diacetic acid (H_4_neunox). A maleimide-containing chelator (H_4_neunox-Mal) was tailored for ^89^Zr radiolabeling of biomolecules. The synthesis involved the reaction of* N-*Succinimidyl 6-maleimidohexanoate with H_4_neunox under basic conditions to produce H_4_neunox-Mal (Figure 7A). Subsequently, H_4_neunox-trastuzumab was synthesized using H_4_neunox-Mal. For comparison, DFO-trastuzumab was synthesized using the corresponding maleimide precursor. Notably, the radiolabeling of H_4_neunox-trastuzumab exhibited slower kinetics with only 91% radiochemical yield after 2 h reaction, whereas DFO-trastuzumab achieved a higher radiochemical yield (>95%) within 1 h. The isolated yields of [^89^Zr]Zr-DFO- and [^89^Zr]Zr-neunox-trastuzumab were 52% and 43%, respectively. Upon subjecting both radiotracers to a serum stability test for 7 days, [^89^Zr]Zr-neunox-trastuzumab displayed greater demetallation, with only 76% of the complex remaining intact compared with the 95% observed for [^89^Zr]Zr-DFO-trastuzumab. PET imaging and biodistribution studies conducted using SKOV-3 cancer xenograft models revealed that [^89^Zr]Zr-DFO-trastuzumab exhibited elevated tumor uptake and minimal bone uptake on days 1 and 4 postinjection. Conversely, [^89^Zr]Zr-neunox-trastuzumab exhibited poor *in vivo* stability, manifesting as high bone uptake at the specified time points (Figure 7B). The biodistribution study corroborated these findings, indicating that [^89^Zr]Zr-neunox-trastuzumab is not suitable for immune PET applications 44.

### [^18^F]AlF-NOTA-MAL

**Figure 8:**
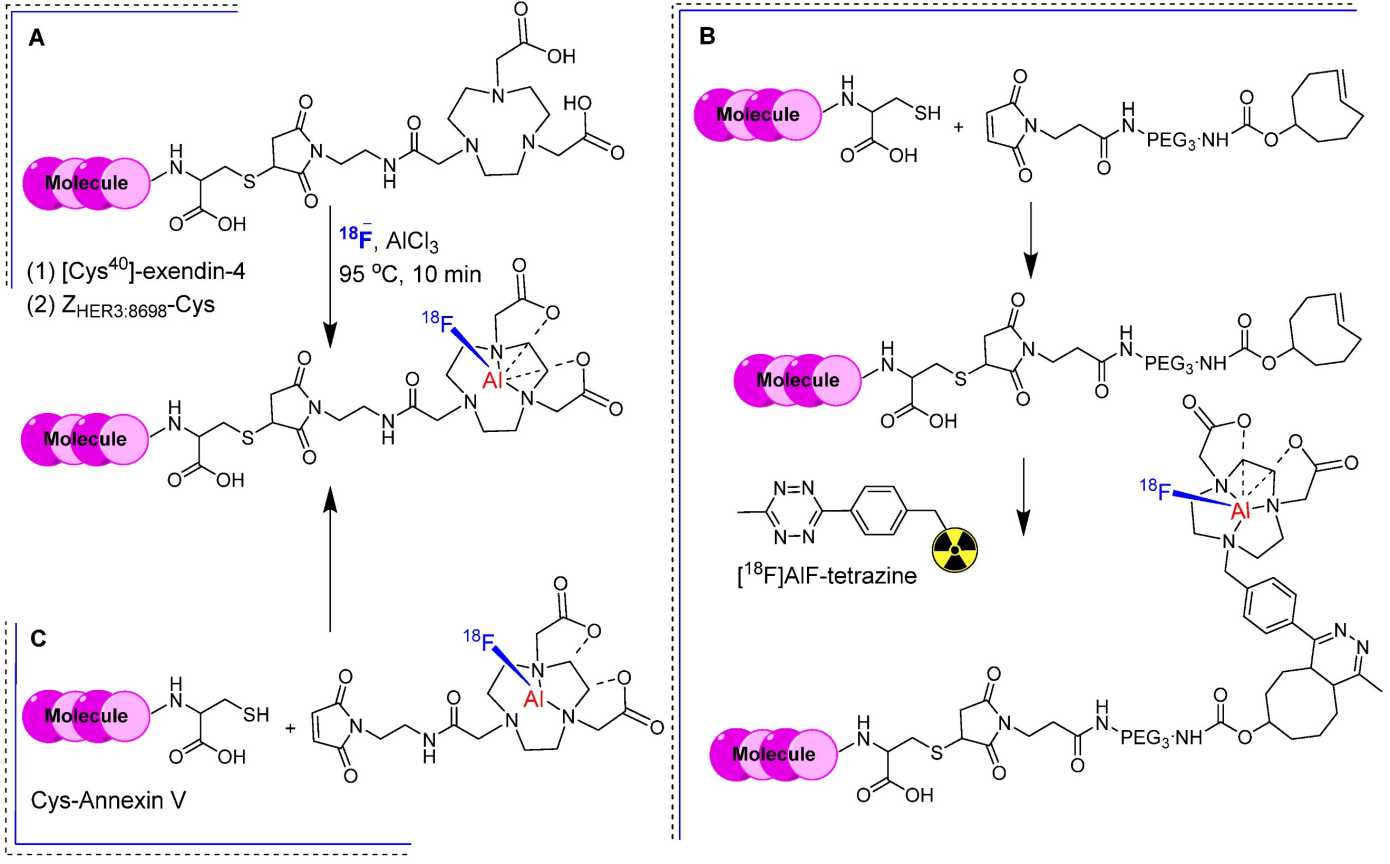
Radiolabeling strategies using NOTA-MAL chelator. (A) Integration of the NOTA-MAL chelator into biomolecules through thiol linkage, followed by radiolabeling utilizing [^18^F]fluoride and AlCl_3_ under mild basic conditions. Exemplary biomolecules include -[Cys^40^]-exendin-4 and Z_HER3:8698_-Cys. (B) Synthesis of TCO-Z_HER3:8698_ and subsequent radiolabeling employing the IEDDA reaction between TCO containing affibody and [^18^F]AlF-tetrazine. (C) Radiolabeling of Cys-annexin V using [^18^F]AlF-NOTA-MAL.

In the previous section, we showed that [^18^F]FBEM-[Cys^40^]-exendin-4 exhibited significant uptake in an insulinoma xenograft model. Nevertheless, its application in imaging insulinoma within the pancreas was constrained by heightened abdominal uptake. To address this limitation, a maleimide-functionalized 1,4,7-triazanonane-1,4,7-triacetate (NOTA-MAL) chelator was synthesized for the radiofluorination of [Cys^40^]-exendin-4 45. Incorporating NOTA-MAL into the [Cys^40^]-exendin-4 peptide resulted in NOTA-MAL-[Cys^40^]-exendin-4, which exhibited notably heightened binding affinity (IC50 = 2.84 nM) compared with that of [Cys^40^]-exendin-4 alone in GLP-1 positive INS-1 cells. The radiolabeling of NOTA-MAL-[Cys^40^]-exendin-4 was performed using [^18^F] fluoride and AlCl_3_ under slightly basic conditions, yielding [^18^F]AlF-NOTA-MAL-[Cys^40^]-exendin-4 at a moderate radiochemical yield (23.6 ± 2.4%) and with robust *in vitro* stability (Figure 8A). PET imaging data collected from an insulinoma (INS-1) xenograft model revealed that [^18^F]AlF-NOTA-MAL-[Cys^40^]-exendin-4 showed elevated tumor uptake (15.7 ± 1.4 %ID/g) and prominent tumor-to-muscle (175.8 ± 26.4) and tumor-to-liver ratios (31.5 ± 6.7) 1 h post-intravenous injection. However, kidney uptake remained high throughout the experiment, albeit reduced in comparison with those of previously reported ^68^Ga or ^111^In analogs 46.

Based on the same theory, Smith *et al.* synthesized a maleimide-functionalized 1,4,7-triazanonane-1,4,7-triacetate (NOTA) and 1,4,7-triazacyclononane-1,4-diacetate (NODA) chelators, respectively, for direct and indirect radiofluorination of bioactive molecules such as the Z_HER3:8698_ affibody. By incorporating NOTA chelator into Z_HER3:8698_-Cys (NOTA-Z_HER3:8698_) via thiol-maleimide chemistry, they produced [^18^F]AlF-NOTA-Z_HER3:8698_ with high radiochemical yield (38.8 ± 5.8%) and purity (>98%) after HPLC purification. Considering the thermal sensitivity of biomolecules, TCO-PEG3-maleimide was used to synthesize TCO-Z_HER3:8698_ under mild conditions. A tetrazine-bearing NODA chelator was synthesized and radiolabeled using an [^18^F]AlF complex to produce [^18^F]AlF-tetrazine. They used the inverse electron demand Diels-Alder (IEDDA) reaction between TCO-containing affibody and ^18^F-labled tetrazine to produce [^18^F]AlF-NODA-Z_HER3:8698_ with high radiochemical yield (70-95%) and purity (>98%) (Figure 8B). Both [^18^F]AlF-NOTA-Z_HER3:8698_ and [^18^F]AlF-NODA-Z_HER3:8698_ exhibited *in vitro* stability of 97.9 ± 0.5% and 91.5 ± 1.2%, respectively. The cell-binding affinities towards HER3 expressing MCF-7 cells were comparable regardless of the conjugation method. Both radiotracers showed high tumor accumulation in MCF-7 tumor-bearing mice, high renal clearance, and significant radioactivity in HER3-expressing organs such as the intestine, liver, and lungs. [^18^F]AlF-NOTA-Z_HER3:8698_ showed higher uptake in the intestine owing to increased lipophilicity, suggesting possible hepatobiliary excretion. Its biodistribution profile and tumor-to-blood (24.61 ± 14.45) and tumor-to-muscle ratios (34.96 ± 8.60) were better than that of [^18^F]AlF-NODA-Z_HER3:8698_,confirming its superiority over IEDDA based conjugation 47. For example, [^18^F]AlF-NOTA-MAL was synthesized at 15% radiochemical yield and used for the radiolabeling of the Cys-annexin V protein (Figure 8C). The resulting [^18^F]-AlF-NOTA-MAL-Cys-annexin V demonstrated robust *in vitro* and *in vivo* stability. This radiotracer has been effectively used to visualize apoptotic animal models using a PET imaging system 48.

### [^18^F]FPenM and [^18^F]FNEM

**Figure 9:**
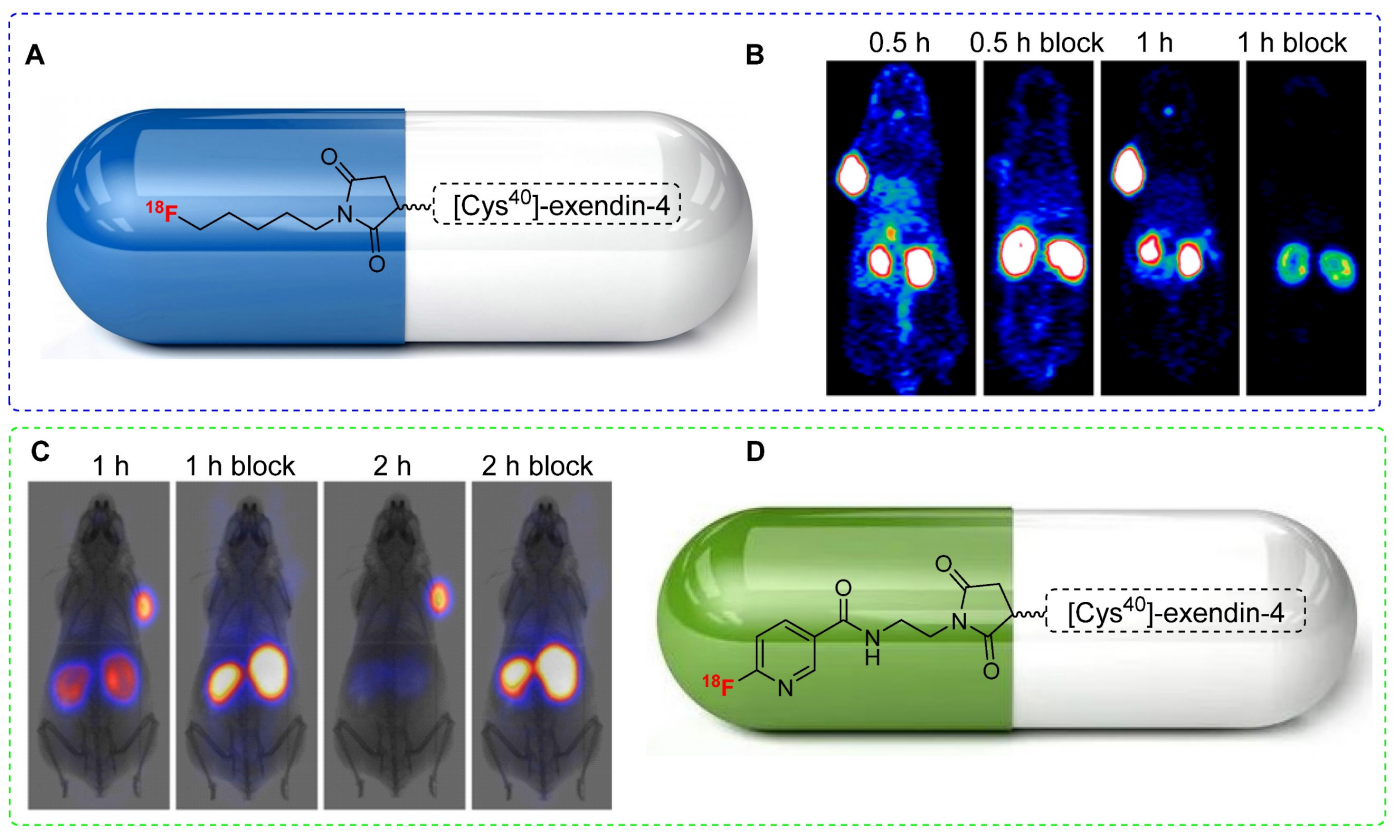
^ 18^F-labeled exendin-4 analogs and PET imaging data. (A) Chemical structure of [^18^F]FPenM-[Cys^40^]-exendin-4. (B) Representative PET images of INS-1 tumor mice at 0.5 and 1 h postinjection of [^18^F]FPenM-[Cys^40^]-exendin-4 under control and blocking conditions, Adapted with permission from 49, copyright 2013, American Chemical Society. (C) Representative PET images of INS-1 tumor mice at 1 and 2 h postinjection of [^18^F]FNEM-[Cys^40^]-exendin-4 under control and blocking conditions. Adapted with permission from 50, copyright 2014, American Chemical Society. (D) Chemical structure [^18^F]FNEM-[Cys^40^]-exendin-4.

Although^18^F-labeled exendin-4 analogs effectively target insulinoma *in vivo*; their practical use is hindered by their non-specific uptake in the abdominal area. To improve pharmacokinetics and minimize abdominal uptake, Chen *et al.* designed a new maleimide-based prosthetic group, N-5-[^18^F]fluoropentylmaleimide ([^18^F]FPenM), featuring a low-molecular-weight aliphatic structure. The synthesis of [^18^F]FPenM involves the use of N-2-[^18^F]fluoropentylamine hydrochloride and N-methoxycarbonylmaleimide under aqueous conditions. Although obtained at a relatively low radiochemical yield (11-17%), [^18^F]FPenM-[Cys^40^]-exendin-4 (Figure 9A) displayed a strong binding affinity to GLP-1. PET imaging using the INS-1 tumor xenograft model showed high tumor uptake (Figure 9B) and substantial tumor-to-background and tumor-to-liver ratios; however, renal uptake remained notably high 49. Despite yielding critical outcomes, [^18^F]FPenM has some limitations such as low radiochemical yield and complex fabrication procedures.

To address these limitations, Chen *et al*. proposed a structural modification by replacing the aliphatic structure with a pyridine ring. N-(2-(2,5-dioxo-2,5-dihydro-1H-pyrrol-1-yl)ethyl)-6-fluoronicotinamide [^18^F]FNEM was synthesized under basic conditions using N-(2-aminoethyl)maleimide trifluoroacetate salt and 6-[^18^F]fluoronicotinic acid 2,3,5,6-tetrafluorophenyl ester. [^18^F]FNEM was obtained at moderate radiochemical yield (26±5%) and high radiochemical purity (>99%). Subsequently, [^18^F]FNEM was conjugated with [Cys^40^]-exendin-4 to produce [^18^F]FNEM-[Cys^40^]-exendin-4 (Figure 9D) at a moderate radiochemical yield (30-40%). PET imaging using [^18^F]FNEM-[Cys^40^]-exendin-4 in INS-1 insulinoma xenograft mice showed tumor uptake comparable to that of [^18^F]FPenM-[Cys^40^]- exendin-4. Notably, this modified prosthetic group exhibited fast renal clearance and high tumor-to-background and tumor-to-liver ratios (Figure 9C) 50.

### ^18^F labeled thiol groups for maleimide containing molecules

**Figure 10:**
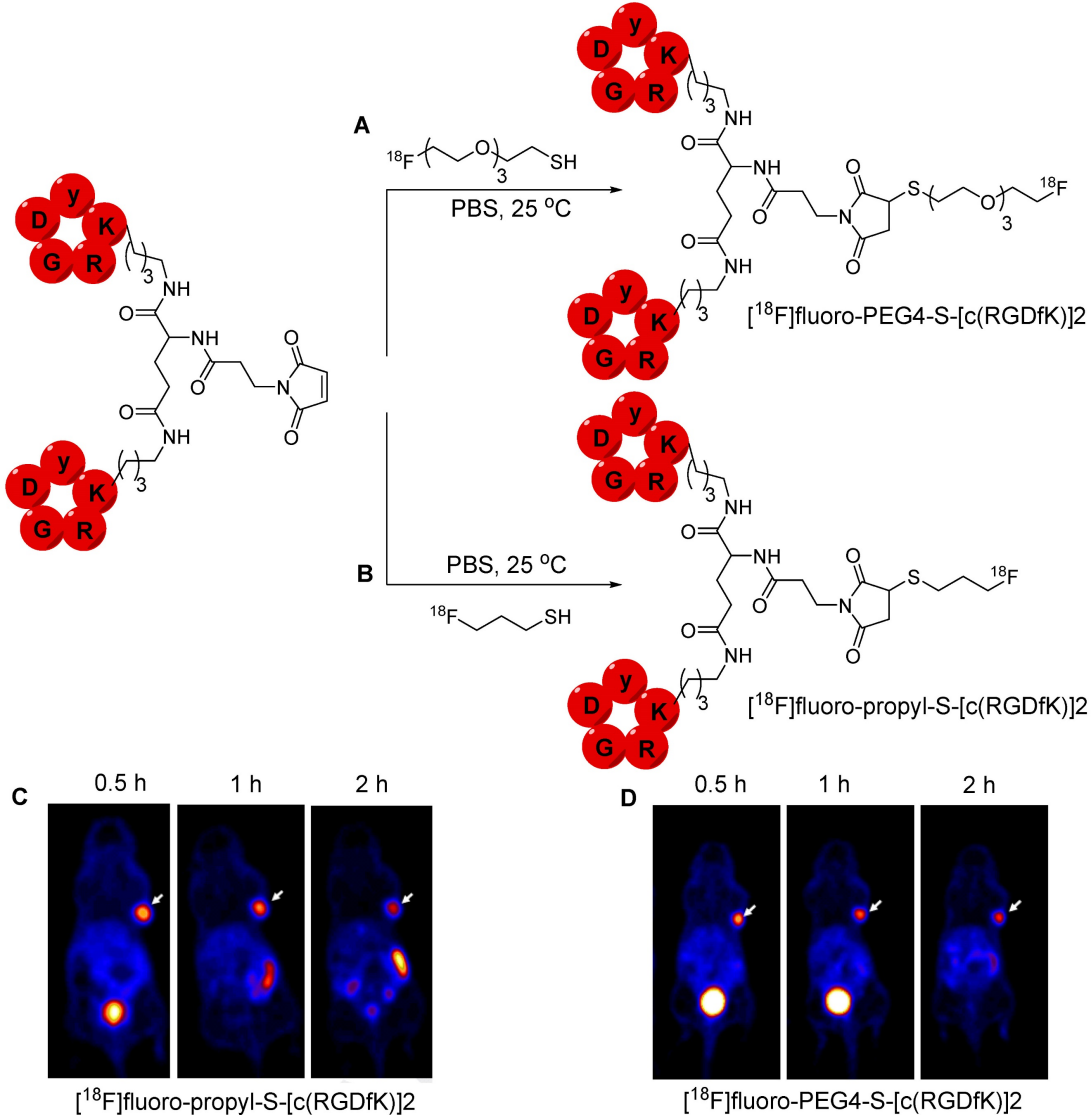
^ 18^F-labeled [c(RGDfK)]2 analogs and PET imaging data. (A) Synthesis of [^18^F]fluoro-PEG4-S-[c(RGDfK)]2 using [^18^F]fluoro-PEG4-thiol. (B) Synthesis of [^18^F]fluoro-propyl-S-[c(RGDfK)]2 using [^18^F]fluoropropyl-thiol. (C) Representative PET images of U87MG tumor mice at 0.5, 1, and 2 h postinjection of [^18^F]fluoro-propyl-S-[c(RGDfK)]2. (D) Representative PET images of U87MG tumor mice at 0.5, 1, and 2 h postinjection of [^18^F]fluoro-PEG4-S-[c(RGDfK)]2. Adapted with permission from 51, copyright 2019, American Chemical Society.

A multistep radiolabeling process is used to counteract the poor stability of the maleimide group under the basic conditions necessary for nucleophilic radiofluorination. This process is cumbersome and diminishes the overall radiochemical yield. In 2019, Jacobson *et al*. proposed an alternative approach using ^18^F radiolabeled thiol-containing precursor. Two prosthetic groups, namely, [^18^F]fluoropropyl-thiol and [^18^F]fluoro-PEG4-thiol were synthesized and used for radiolabeling maleimide-containing biomolecules under physiological conditions. [^18^F]fluoropropyl benzothioate and [^18^F]Fluoro-PEG4 were obtained in a moderate radiochemical yield of 37-47% and 28-35%, respectively. Benzothioate facilitates efficient protection and rapid deprotection using sodium methoxide, followed by a conjugation reaction in the same vessel. The radiolabeling efficiency of [^18^F]fluoropropyl-thiol and [^18^F]fluoro-PEG4-thiol was assessed using a maleimide-containing molecules such as Evans Blue, a PSMA analog, and the [c(RGDfK)]2 peptide (Figure 10A-B). The ^18^F-labeled compounds showed high *in vitro* stability in PBS and mouse serum. Moreover, ^18^F-labled peptides were subjected to further *in vivo* studies. PET imaging data from the U87MG tumor model revealed the rapid clearance of both [^18^F]fluoro-PEG4-S-[c(RGDfK)]2 and [^18^F]fluoro-propyl-S-[c(RGDfK)]2 from background tissues and high accumulation in the tumor. However, the PEG4-based tracer showed superior pharmacokinetics while exhibiting minimal accumulation in the intestine over time owing to its enhanced hydrophilic nature (Figure 10C-D) 51. Based on these results, Schirrmacher *et al*. proposed ^18^F labeling of maleimide-containing biomolecules such as rat serum protein. To achieve this, a maleimide group was incorporated into the protein using 4-(N-maleimidomethyl)cyclohexane-1-carboxylic acid 3-sulfo-N-hydroxy-succinimide ester sodium salt (sulfo-SMCC). The silicon fluoride acceptor reagent [^18^F]SiFA-SH was synthesized using an isotopic exchange reaction, which was subsequently used for radiolabeling the protein with the installed maleimide group. Nevertheless, the radiolabeled compound was not subjected to further studies 52. This radiolabeling method involves the incorporation of a maleimide group using a lysine functional group. Although this approach appears suitable for radiolabeling peptides, it may pose challenges when applied to macromolecules because of the potential for nonspecific radiolabeling of molecules similar to that encountered with other lysine-based radiolabeling approaches.

**Figure 11:**
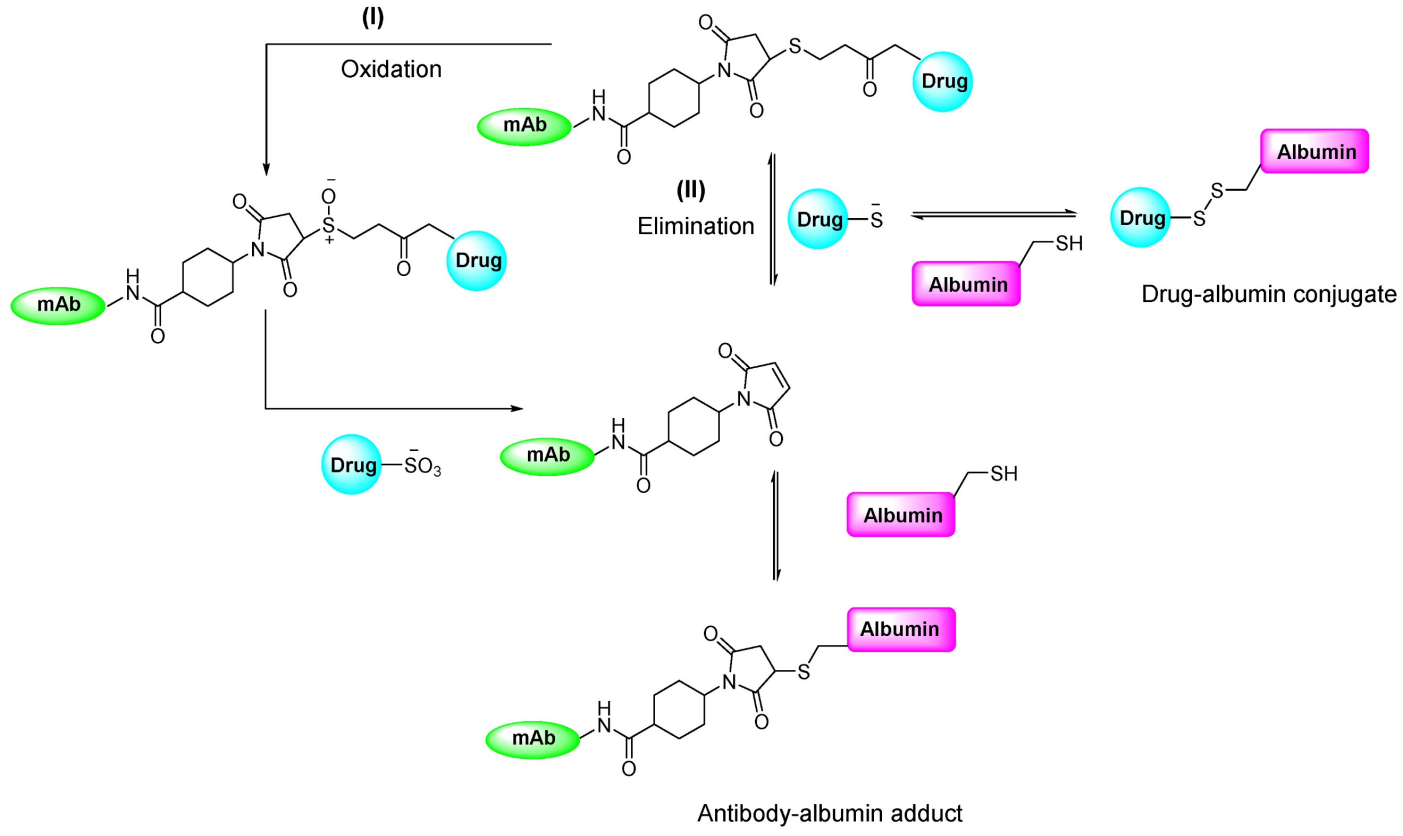
Proposed mechanism elucidating the *in vivo* instability of thiosuccinimide adducts under physiological conditions. Two mechanisms have been posited to account for the cleavage of the resulting product: (I) retro-Michael β-elimination reaction, and (II) oxidative cleavage of the product 53.

Overall, maleimide-based prosthetic groups remain the preferred option for cysteine-based radiolabeling of biomolecules, owing to their distinct properties that are outlined in Table 1. Maleimide-thiol-based modifications are recognized for their rapid kinetics and achieving quantitative and highly specific reactions under physiological conditions. However, maleimide-thiol adducts are notably unstable under physiological conditions owing to thiol exchange and retro-Michael addition reactions (Figure 11). Pharmacologically, the instability of thiosuccinimide adducts and premature cleavage of therapeutic or diagnostic radionuclides lead to reduced drug cytotoxicity, heightened off-target toxicity, or a decline in imaging quality. Moreover, the hydrolysis of thiosuccinimide and subsequent ring-opening pose additional challenges.

## Vinyl sulfone-based radiolabeling

Vinyl sulfone consists of a vinyl moiety that is covalently bonded to an electrophilic sulfone group. They act as building blocks in various organic syntheses and as crucial structural components in various biologically important molecules and drugs. Essentially, diaryl vinyl sulfone represents a structural derivative of the chalcone-like structure, when the α,β- unsaturated carbonyl moiety is replaced by vinyl sulfone 54. The vinyl sulfone functional group is important for amine- or thiol-based conjugation with macro-biomolecules such as proteins. Vinyl sulfone can specifically conjugate with free thiol groups and avoid nonspecific reactions with other nucleophilic functional groups such as amines or histidine under mildly acidic conditions 55. Vinylsulfone-based radiolabeled prosthetic groups exhibit higher stability than that of their maleimide-based counterparts, which are prone to hydrolysis. The resulting adduct was stable both *in vitro* and *in vivo*.

### Radiometal chelator-based vinyl sulfone

**Figure 12:**
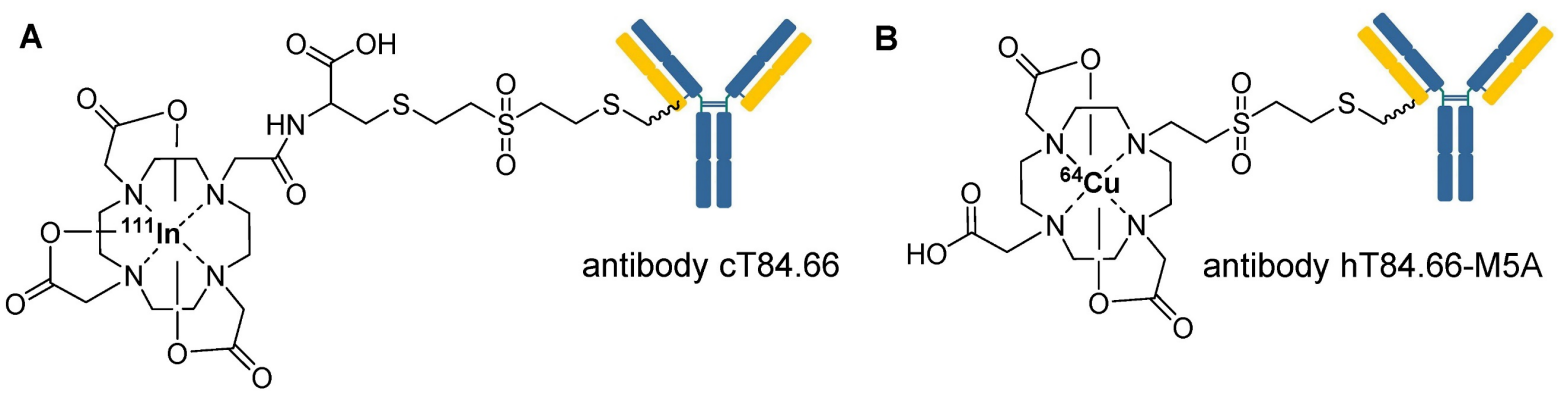
Representative chemical structures of radiolabeled biomolecules utilizing vinyl sulfone-based prosthetic group, (A) ^111^In-labeled chimeric anti-CEA antibody cT84.66, and (B) ^64^Cu-labeled humanized anti-CEA antibody hT84.66-M5A (M5A).

The potential of a vinyl sulfone-based bifunctional chelating agent (BCA) for the synthesis of radiopharmaceuticals was explored. Initially, a maleimide-based DOTA chelator was used to radiolabel the monoclonal antibody. However, pH-dependent cleavage of the resulting bioconjugate was observed over time, leading to a high blood clearance rate, which ultimately hindered effective tumor uptake 56, 57. In a modified approach, a vinyl sulfone-based prosthetic group was synthesized using a DOTA chelator. This novel prosthetic group was used for radiolabeling the chimeric anti-CEA antibody cT84.66 with indium-111 (^111^In) (half-life, 2 days) (Figure 12A). Despite concerns regarding the reaction of vinyl sulfone with amino groups, nonspecific radiolabeling was effectively controlled by manipulating the pH of the reaction mixture. The same prosthetic groups react with amino groups at pH = 9 in the absence of a reducing agent. However, the reaction towards the sulfhydryl group was dominant at neutral pH in the presence of a reducing agent. Comparative biodistribution using human colon tumor xenograft models revealed superior tumor-to-blood (T/B) and tumor-to-liver (T/L) ratios for SH-specific conjugates compared with that of the NH-based conjugates. This difference was attributed to the slow blood clearance and comparatively higher tumor uptake observed for SH-specific conjugates 58.

In a subsequent investigation, Shively *et al*. radiolabeled the humanized anti-CEA antibody hT84.66-M5A (M5A) with ^111^In or ^64^Cu using vinylsulfone-DOTA- or DOTA-NHS-based BCAs. The vinyl sulfone-DOTA conjugate (Figure 12B), regardless of whether thiol or amine linkage was used, exhibited high *in vivo* stability and minimal liver uptake compared with that of the commercially available DOTA-NHS ester 59.

### ^18^F-DEG-VS and ^18^F-PEG_n_-VS

**Figure 13:**
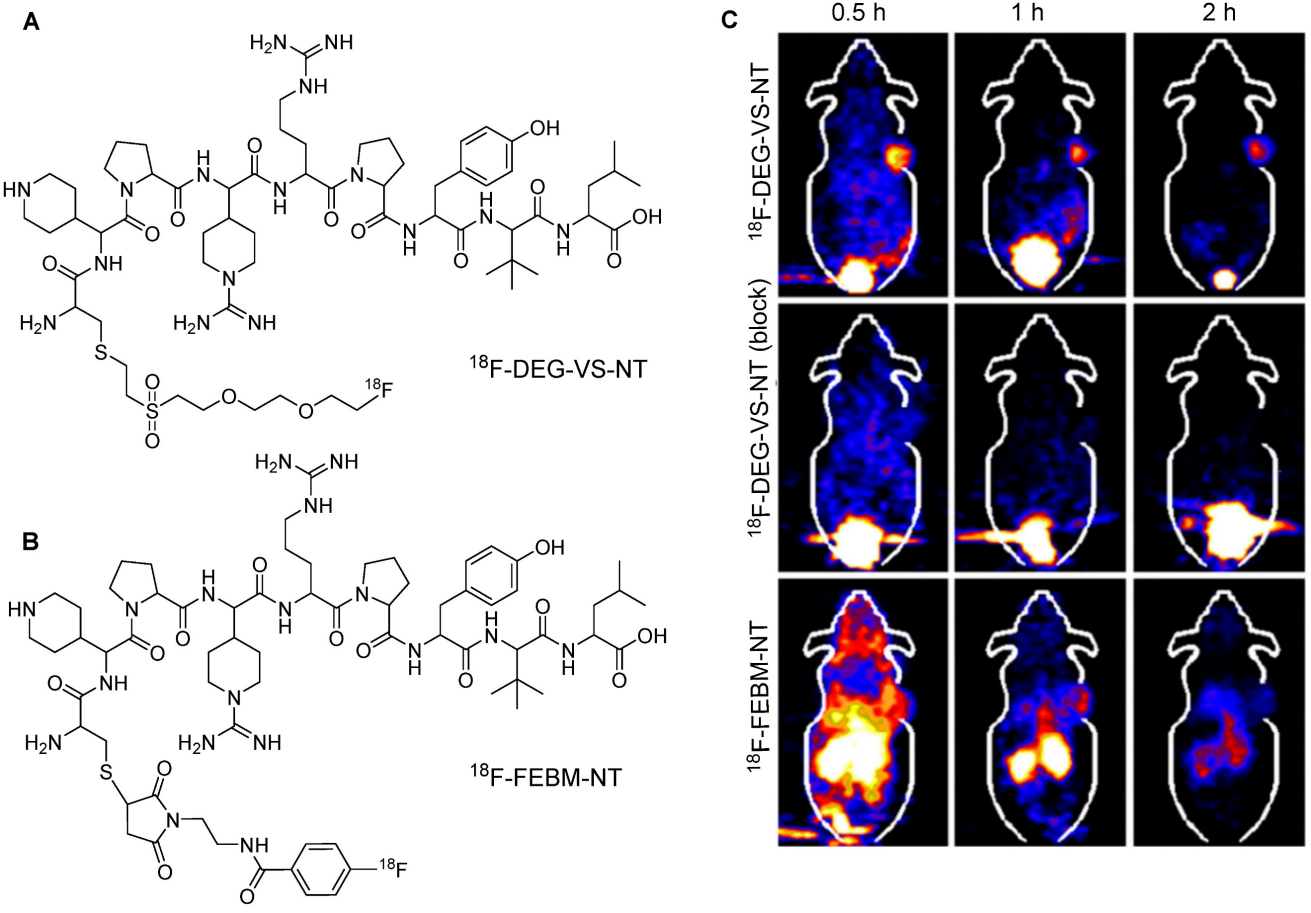
^18^F-labeling of thiolated neurotensin (NT) peptide and *in vivo* imaging study. (A) Chemical structure of ^18^F-DEG-VS-NT peptide. (B) Chemical structure of ^18^F-FBEM-NT peptide. (C) PET imaging data for ^18^F-DEG-VS-NT peptide (under normal or blocking conditions) and ^18^F-FBEM-NT peptide utilizing HT-29 xenograft model. Adapted with permission from 60, copyright 2014, The Society of Nuclear Medicine and Molecular Imaging, Inc.

The use of a PEG linker has been reported for the synthesis of ^18^F-labeled vinyl sulfone-based prosthetic groups, specifically that of (2-(2-(2-[^18^F]fluoroethoxy)ethoxy)ethylsulfonyl)ethane (^18^F-DEG-VS), for the radiolabeling of thiol-containing biomolecules. The application of ^18^F-DEG-VS in the radiolabeling of c(RGDyC), c(RGDyK)_2_, and thiolated neurotensin (NT) peptides was investigated 60. This study revealed that^ 18^F-DEG-VS efficiently reacted with the free thiol of c(RGDyC) within 30 min at neutral pH, resulting in a high radiochemical yield. However, the reaction with the free amino group of c(RGDyK) provided moderate radiochemical yield under basic conditions after 24 h of incubation. ^18^F-labeled NT peptide (^18^F-DEG-VS-NT) was obtained at a substantial radiochemical yield. Both radiolabeled and non-modified NT peptides showed similar binding affinities towards NTR1-positive HT-29 cells. PET imaging data were acquired using the HT-29 xenograft model and the targeting efficiency ^18^F-DEG-VS-NT (Figure 13A) was compared with that of maleimide-based ^18^F labeled NT peptide (^18^F-FBEM-NT) (Figure 13B). Compared with that of the maleimide-based precursor, ^18^F-DEG-VS-NT exhibited superior tumor targeting, lower accumulation in abdominal tissues, and higher tumor-to-background contrast (Figure 13C).

**Figure 14:**
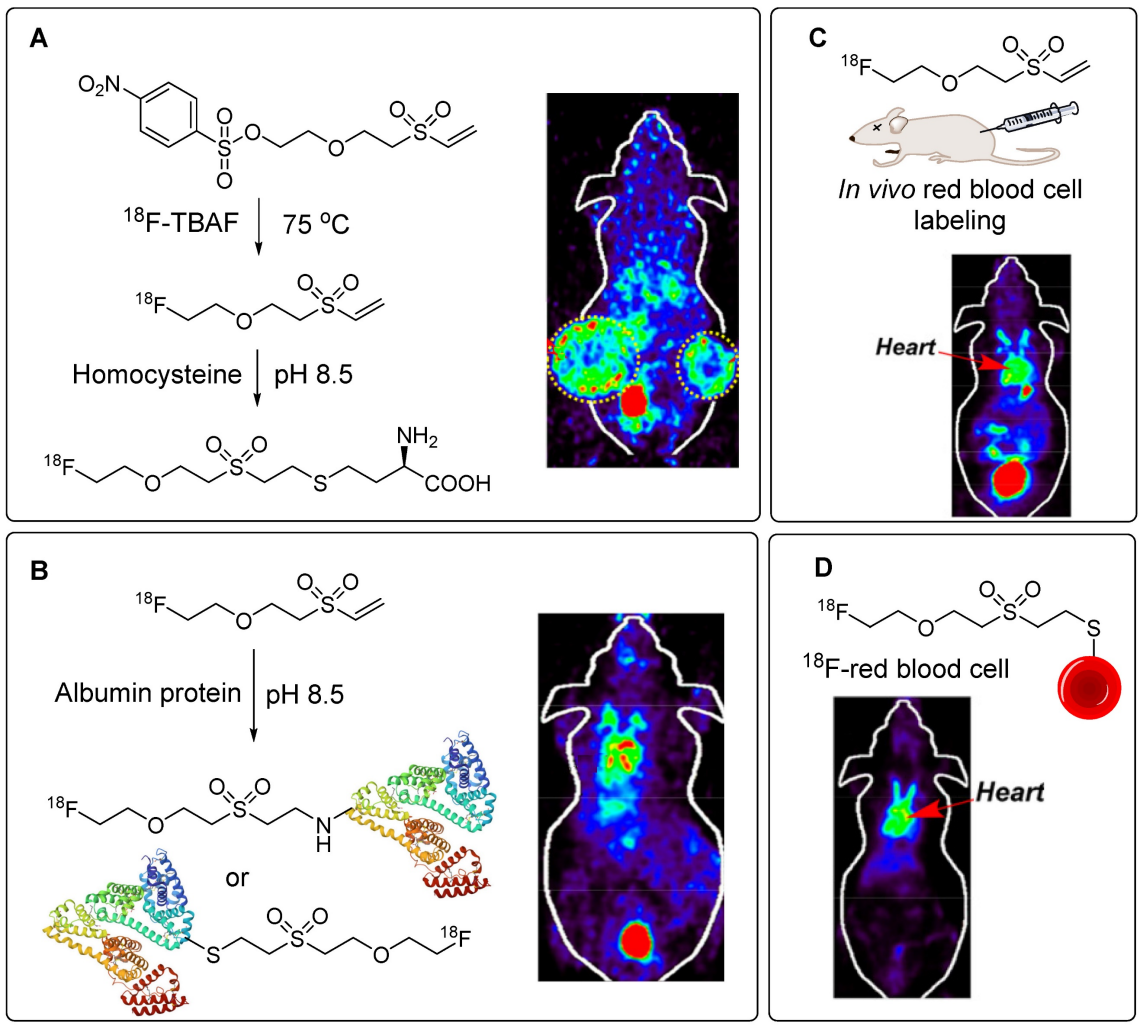
^18^F-labeling of thiol-containing biomolecules utilizing ^18^F-PEG_1_-VS for *in vivo* imaging study. (A) ^18^F-labeling of homocysteine and PET imaging utilizing H1299 tumor model. (B) ^18^F-labeling of albumin protein and PET imaging of blood pool. (C) *In vivo* radiolabeling of red blood cell utilizing ^18^F-PEG_1_-VS and PET imaging of cardiac area (D)* In vitro* radiolabeling of red blood cell utilizing ^18^F-PEG_1_-VS and PET imaging of cardiac area. Adapted with permission from 61, copyright 2020, American Chemical Society.

Further analysis of the potential of this prosthetic group showed that the addition of a PEG linker could significantly enhance its biomedical application. Accordingly, three compounds, namely, ^18^F-PEG_1_-VS, ^18^F-PEG_2_-VS, and ^18^F-PEG_3_-VS, were synthesized using their corresponding nosylate precursors, for a moderate radiochemical yield (19-26%). ^18^F-PEG_1_-VS was optimized for thiol- or amine-based radiolabeling of biomolecules. The prosthetic group provided moderate to high radiochemical yields of 13-97% and 7-58%, respectively, within 30 min at pH = 8.5 and room temperature. ^18^F-PEG_1_-VS was used to radiolabel homocysteine-SH, GSH, glucose-SH, and coenzyme-SH using thiol-based linkages. Similarly, triphenyl phosphate-NH_2_ (TTP2 and TTP6), cRGDyK, and mouse serum protein were radiolabeled using amine-based linkages. Homocysteine-SH was radiolabeled with >95% radiochemical yield and effectively targeted the H1299 tumor model *in vivo* (Figure 14A). Similarly, the ^18^F-radiolabeled TTP-NH_2_, cRGDyK peptide, and mouse serum proteins were obtained at quantitative radiolabeling yields and were used for PET imaging of the cardiac area, U87MG tumors, and blood pool imaging (Figure 14B), respectively. Notably, all three prosthetic groups were used to radiolabel red blood cells *in vivo*, demonstrating their potential applications in gastrointestinal bleeding scintigraphy (GIBS) (Figure 14C). All three prosthetic groups were intravenously injected into mice. Blood pool activity revealed that ^18^F-PEG_1_-VS and ^18^F-PGE_2_-VS showed similar pharmacokinetics and high uptake in the heart with minimal background. However, ^18^F-PEG_3_-VS showed high cardiac uptake but poor imaging contrast. Additionally, ^18^F-PEG_1_-VS efficiently radiolabeled red blood cells *in vitro* with a high radiochemical yield (75%) under mildly basic conditions (Figure 14D). The radiolabeled red blood cells (^18^F-PEG_1_-VS-RBCs) were efficiently used for PET imaging of extravascular blood in the abdominal region and the cardiovascular system. The radiotracer exhibited prolonged residence within the cardiac blood pool, with its uptake remaining unaffected by variations in blood glucose levels 61.

**Figure 15:**
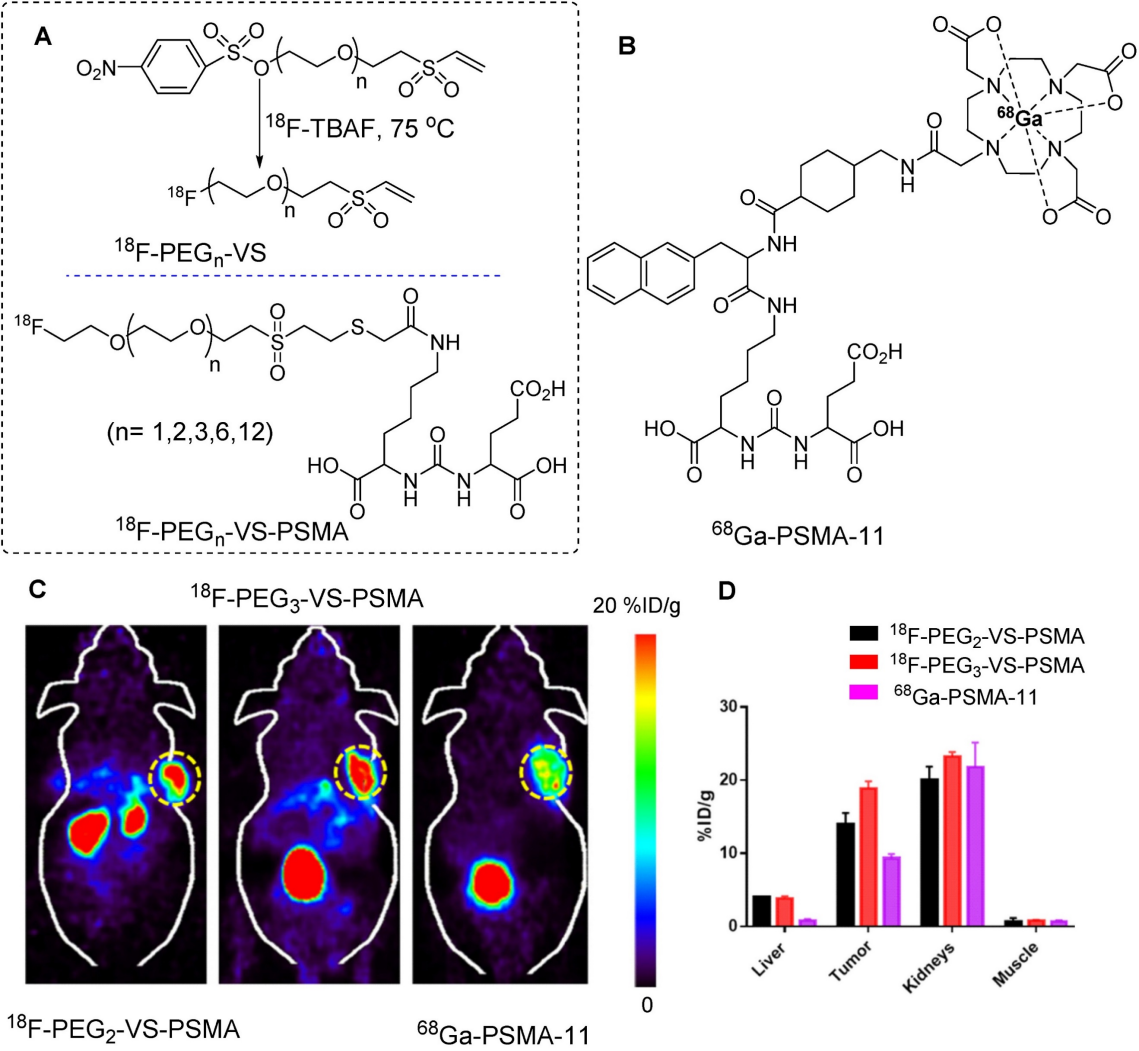
^18^F-labeling of thiol-containing PSMA molecule and *in vivo* imaging study. (A) The ^18^F-labeling of thiol-containing PSMA molecule was conducted using ^18^F-PEG_n_-VS prosthetic group. (B) Chemical structure of clinically approved ^68^Ga-PSMA-11 molecule. (C) PET imaging and (D) biodistribution data comparison study for ^18^F-(PEG)_2_-VS-PSMA, ^18^F-(PEG)_3_-VS-PSMA, and ^68^Ga-PSMA-11 utilizing the LNCaP tumor model (Data were acquired 0.5 h post injection). Adapted with permission from 63, copyright 2021, American Chemical Society.

In another study, red blood cells were radiolabeled with the same prosthetic group for detailed *in vivo* analysis. Notably, ^18^F-PEG_1_-VS crossed the blood-brain barrier and accumulated in the cerebellum. The uptake of ^18^F-PEG_1_-VS is presumed to be related to the presence of free thiol groups in brain tissues. This suggests that brain imaging can be performed using this precursor 62. Using a similar approach, Wu *et al.* synthesized a series of PSMA-targeting tracers using ^18^F-(PEG)_n_-VS prosthetic groups (Figure 15A). The incorporation of a PEG_n_ linker (where n=1, 2, 3, 6, and 12) into the main structure was aimed at enhancing hydrophilicity. Consequently, improved tumor-to-background contrast was achieved. Binding affinity, pharmacokinetics, and PET imaging data were acquired using PSMA-positive LNCaP animal models, and the results were benchmarked against the clinically approved ^68^Ga-PSMA-11 precursor (Figure 15B). Notably, ^18^F-(PEG)_2_-VS-PSMA and ^18^F-(PEG)_3_-VS-PSMA exhibited higher binding affinities for PSMA-positive C4-2B cells than with their counterparts. PET imaging and organ distribution data in LNCaP tumors revealed elevated tumor uptake for (PEG)_2_ and (PEG)_3_, which registered at 8.8 ± 3.3 and 12 ± 2.2 %ID/g at 0.5 h, respectively. Moreover, this heightened uptake persisted for up to 3 h. Notably, the tumor-to-muscle ratio of ^18^F-(PEG)_1_-VS-PSMA surpassed those of (PEG)_2_ and (PEG)_3_ owing to the shortness of the PEG chain and rapid washout from the muscles. The radiolabeled precursor demonstrated high specificity, and blocking caused a significant reduction in both tumor and kidney uptake. Notably, the tumor uptake of ^18^F-(PEG)_2_-VS-PSMA and ^18^F-(PEG)_3_-VS-PSMA was nearly double that of the clinically approved ^68^Ga-PSMA-11 (Figure 15C-D), whereas comparable uptake was maintained in the muscles and kidneys 63.

### [^18^F]FVSB

**Figure 16:**
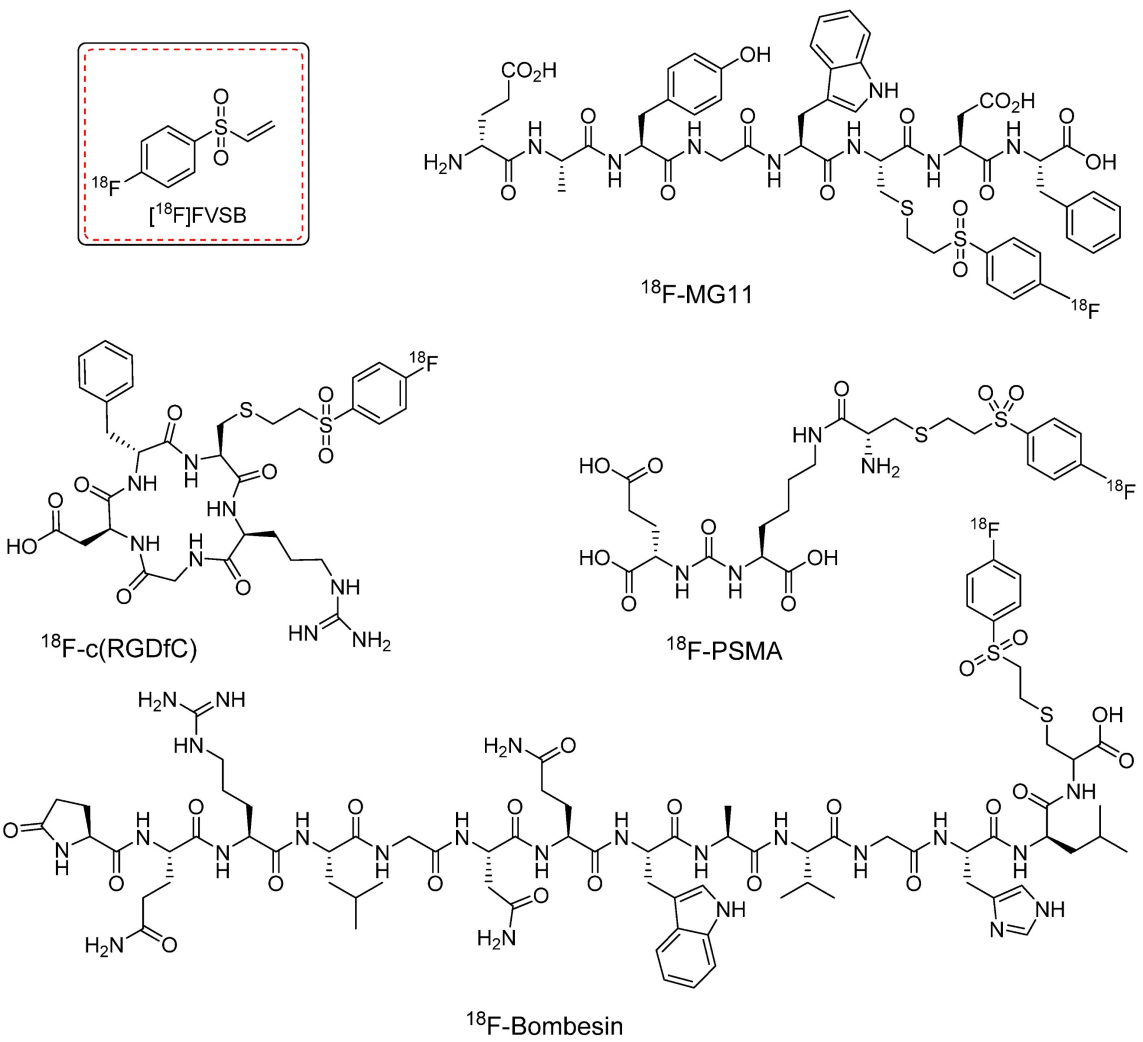
Chemical structures of clinically approved ^18^F-labeled peptides. These peptides were radiolabeled using [^18^F]fluoro-4-(vinylsulfonyl)benzene ([^18^F]FVSB) prosthetic group.

^18^F-PEG_n_-VS and its derivatives are recognized for their radiolabeling efficiencies. However, despite notable advancements, the synthesis of these prosthetic groups traditionally involves azeotropic drying and HPLC purification steps before the thiol conjugation reaction. This multistep synthesis process and the potential instability of the precursors often result in low radiochemical yields. To address these challenges, Murphy *et al.* introduced a novel prosthetic group, namely, [^18^F]fluoro-4-(vinylsulfonyl)benzene ([^18^F]FVSB), which was designed for radiolabeling of thiol-containing biomolecules 64. The synthesis of [^18^F]FVSB used a highly stable uranium-based intermediate precursor, which eliminates the need for additional steps such as azeotropic drying or HPLC purification. [^18^F]FVSB was synthesized within 30 min at 130 °C with high radiochemical yield (46 ± 4%, decay corrected) and purity (85%). The radiofluorinated precursor was directly used for the thiol-based radiolabeling of biologically active peptides. [^18^F]FVSB exhibited excellent radiolabeling efficiency under physiological conditions when used to radiolabel clinically important peptide analogs, including the linear RGD peptide (RCY = 84 ± 8%), cyclic (RGDfC) peptide (RCY = 87 ± 2%), PSMA analogue (RCY = 80 ± 3%), MG11 analogue (RCY = 83 ± 10%), neuromedin B analogue (RCY = 93 ± 1%), and bombesin analogue (RCY = 55 ± 11%) (Figure 16). However, this study did not include *in vitro* or *in vivo* investigations.

**Figure 17:**
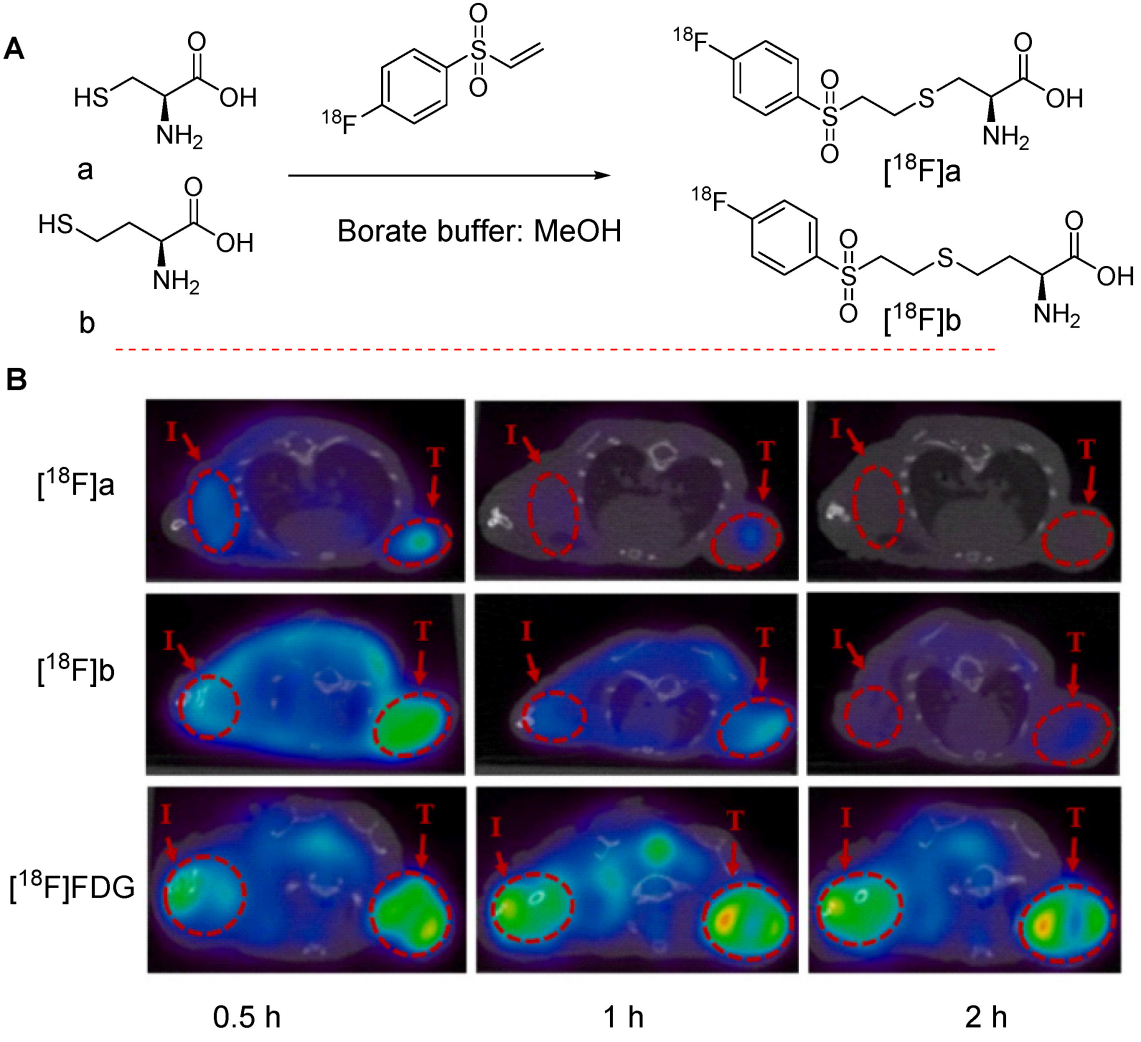
Chemical structures of clinically important ^18^F-labeled amino acids and *in vivo* imaging data. (A) Synthesis of ^18^F-labeled L-cysteine and L-homocysteine molecules, denoted as [^18^F]a and [^18^F]b, respectively (B) PET imaging of [^18^F]a and [^18^F]b compared with [^18^F]FDG in NCI-H1975 tumor-bearing mice with inflammation introduced in the shoulder. Tumor and inflammation regions are denoted as T and I, respectively. Adapted with permission from 65, copyright 2022, Elsevier Inc.

Recently, Mei *et al.* investigated the potential of [^18^F]FVSB to aid the synthesis of ^18^F labeled amino acid analogs intended for PET/CT imaging in inflammation and tumor-bearing animal models. In this study, various amino acids, and derivatives such as L-cysteine, L-homocysteine, D-cysteine, and the small peptide glutathione were radiolabeled to produce the corresponding ^18^F labeled precursors. A series of initial tests including stability assessment, cancer cell uptake testing, specificity evaluation, biodistribution analysis, and PET/CT imaging were performed. The results revealed that the radiolabeled precursors corresponding to L-cysteine and L-homocysteine, denoted as [^18^F]a and [^18^F]b, respectively, were shortlisted as suitable candidates for PET imaging of tumors and inflammatory lesions (Figure 17A). Both precursors exhibited high uptake in the tumor (T) and inflammatory regions (I) of mice bearing NCI-H1975 tumors and inflammation in the shoulder. However, the uptake values were lower than those observed with [^18^F]FDG 65 (Figure 17B).

Despite obtaining encouraging radiolabeling results with vinyl sulfone analogs, concerns persist regarding their reaction with amino groups, leading to nonspecific radiolabeling. Additionally, slow reaction kinetics further emphasizes the need for research to explore better and more efficient thiol-specific radiolabeling prosthetic groups.

## Perfluoroarylation based radiolabeling

**Figure 18:**
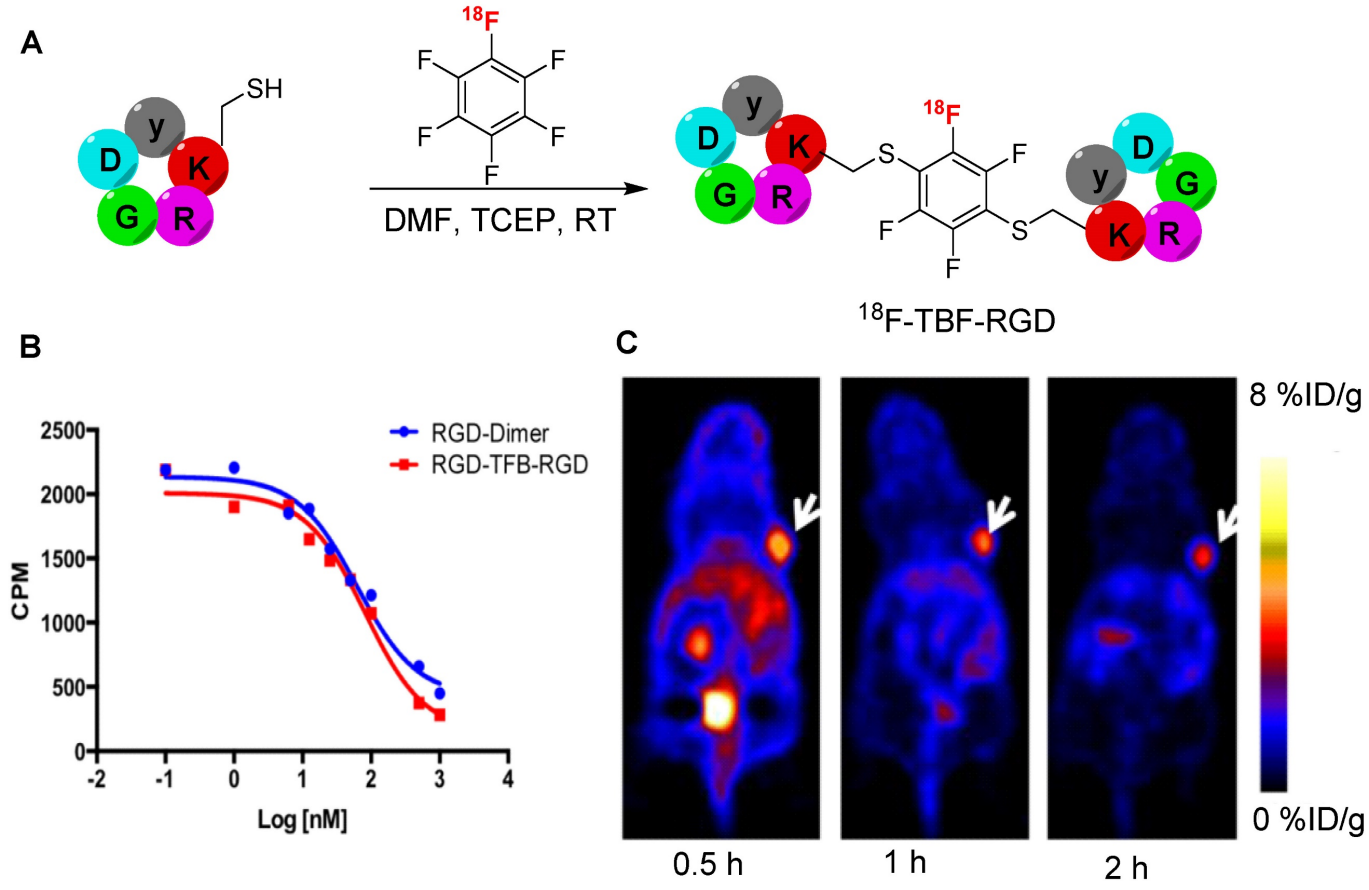
Synthesis of ^18^F-TFB-RGD peptide and *in vitro* and *in vivo* studies. (A) Synthesis of ^18^F-TFB-RGD using [^18^F]HFB prosthetic group. (B) Competition cell binding assay of RGD-TFB-RGD and c(RGDfK)-dimer against ^125^I-echistation in U87MG α_v_β_3_ expressing cells. (C) Representative PET images of ^18^F-RGD-TFB-RGD using U87MG tumor bearing mouse (tumor marked with white arrows) at 0.5, 1, and 2 h postinjection. Adapted with permission from 69, copyright 2015, American Chemical Society.

Pentelute *et al.* were the first to report an aromatic nucleophilic substitution involving sulfhydryl groups in small proteins, peptides, and hexafluorobenzene (HFB) molecules. Subsequently, HBF was used to exclusively staple various peptides, each featuring a free thiol group, via 1,4-disubstitution 66. Although some genetic modifications are necessary to apply this strategy to proteins, this methodology has been extensively used in peptide synthesis, particularly in the context of macrocyclization 67. The yield of this reaction was lower than that of other thiol-based substitution reactions owing to the poor solubility in water and slow reactivity 68. Chen *et al*. explored the potential of HFB in radiolabeling chemistry. HFB was radiolabeled via fluorine exchange reaction using [^18^F]KF-K_2.2.2_ complex in DMSO to obtain [^18^F]HFB at high radiochemical yield (25 ± 3%) and purity (>99%) under optimized conditions. The radiolabeling potential of [^18^F]HFB was tested on thiol containing c(RGDfK) peptide, yielding the dimeric ^18^F-tetrafluorobenzene-RGD (^18^F-TFB-RGD, Figure 18A) at high radiochemical yield (40 ± 2%). The cell binding affinity of ^18^F-TFB-RGD in U87MG α_v_β_3_ expressing cells (IC_50_ = 81 nM) was comparable to that of dimeric c(RGDfK) peptide (IC_50_ = 62 nM) (Figure 18B). PET using ^18^F-TFB-RGD in the U87MG xenograft model showed high tumor uptake (4 %ID/g, 1 h postinjection) and a high T/M ratio (9.8) (Figure 18C). These results are comparable to those of the dimeric c(RGDfK) peptide radiolabeled with *N*-succinimidyl 4-[^18^F]-fluorobenzoate. The tumor specificity of ^18^F-TFB-RGD was confirmed through blocking studies. Moreover, no defluorination or ^18^F uptake was observed in the bone for up to 2 h postinjection. These findings suggest that [^18^F]HFB is a highly specific and stable prosthetic group that enables the radiolabeling of clinically significant peptides and generation of corresponding cyclic or dimeric products 69. Further studies are anticipated to explore the full potential of radiolabeled HBF prosthetic groups, particularly in the context of radiolabeling proteins and antibodies.

## Aryl sulfone based radiolabeling

The limitations associated with the addition of maleimides have prompted researchers to design rapid and highly efficient thiol-specific reactions. Initially, functionalized sulfone reagents were investigated for thiol blocking, and their applicability was subsequently extended to drug conjugation and radiolabeling of biomolecules 70. For instance, Zhang *et al*. studied a series of thiol-blocking reagents and found that 2-(methanesulfonyl)benzothiazole (MSBT) is a novel thiol-specific blocking agent with high reactivity and selectivity 71. Nevertheless, the chemistry of methylsulfonyl-functionalized heteroaromatic compounds and their reactivity towards thiols remain unexplored. In a separate investigation, Toda *et al.* explored a new class of thiol-reactive heteroaromatic methylsulfones for thiol-based conjugation of proteins and peptides. Within this category, phenyloxadiazole methylsulfone (POS) was identified as the most efficient prosthetic group as it demonstrated a thiol-specific addition reaction within a few minutes in aqueous media at pH = 7.0 and room temperature 72. Subsequently, this compound has been used in radiopharmaceutical development.

### ^18^F-FPOS and ^125^I-MSTP

**Figure 19:**
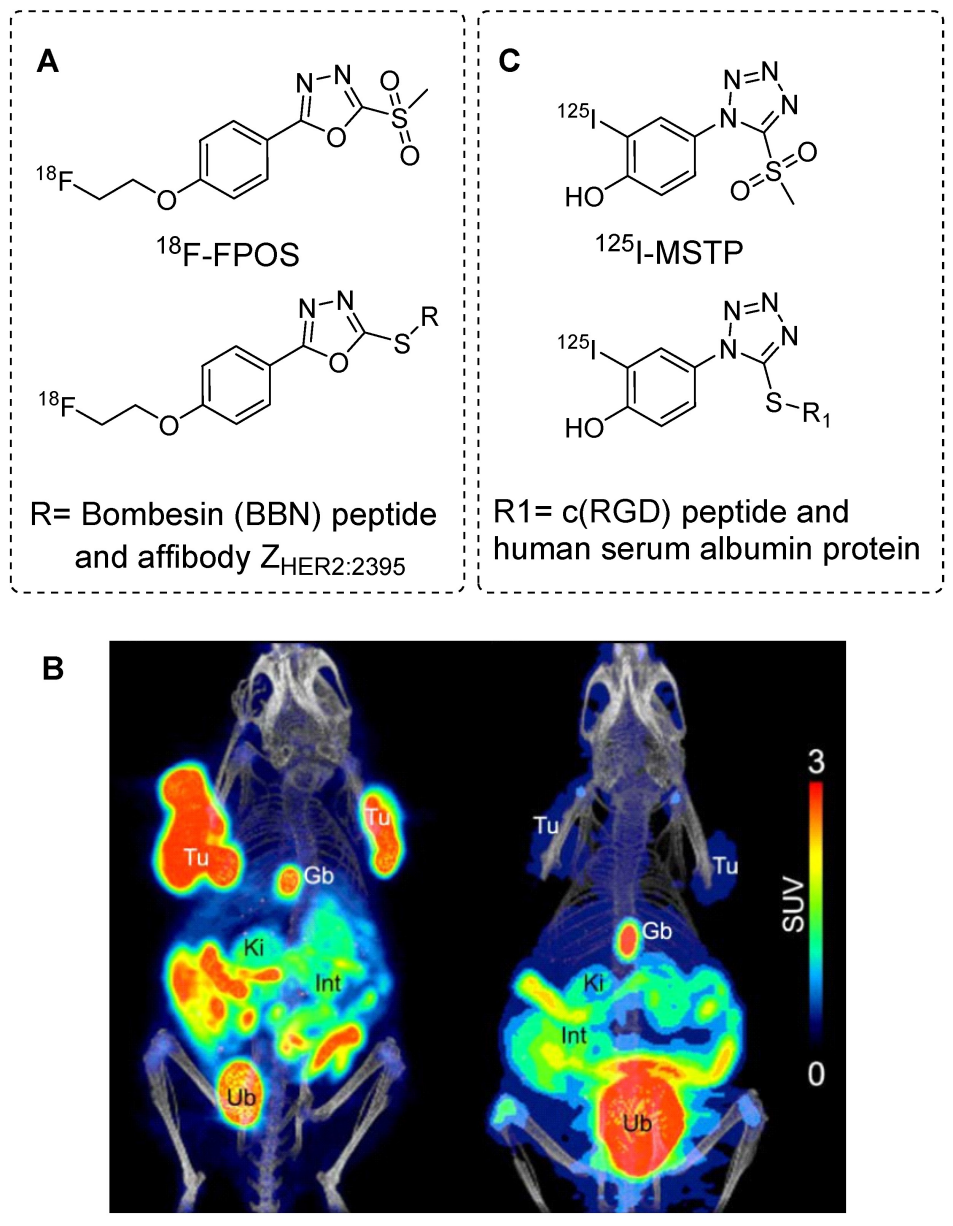
Aryl sulfones based radiolabeling of biomolecules and *in vivo* studies. (A) Chemical structure of ^18^F-FPOS prosthetic group and its corresponding radiolabeled conjugates. (B) Representative PET/CT images performed with HER2‐positive SKOV3 (left side) and HER2‐negative RAMOS (right side) tumor‐bearing mice 2 h p.i. of ^18^F-FPOS-Z_HER2:2395_. Tu: tumors; Gb: gall bladder; Ki: kidneys, Int: intestine; Ub: urinary bladder. Adapted with permission from 73, copyright 2016, The Royal Society of Chemistry. (C) Chemical structure of ^125^I-labeled MSTP prosthetic group and its corresponding radiolabeled conjugates.

The radiolabeling of phenyloxadiazole methylsulfone (POS) with ^18^F was initially performed to explore the potential applications of POS in radiopharmaceutical development. A nosylated phenyloxadiazole methylsulfone derivate was synthesized using commercially available methyl 4‐hydroxybenzoate. This nosylate derivative was radiolabeled with ^18^F using the [^18^F]KF-K_2.2.2_ complex to obtain the corresponding [^18^F]fluoro phenyloxadiazole methylsulfone (^18^F-FPOS) at a moderate radiochemical yield (27 ± 6%) and high radiochemical purity. Then, the efficacy of^ 18^F-FPOS was assessed by radiolabeling clinically important biomolecules such as bombesin (BBN) and the affibody Z_HER2:2395_ (Figure 19A). ^18^F-FPOS-BBN and ^18^F-FPOS-Z_HER2:2395_ were obtained at substantial radiochemical yields of 33% and 40%, respectively, within a short period of 15 min under physiological conditions.^18^F-FPOS-Z_HER2:2395_ was isolated using a size-exclusion cartridge, and its tumor-targeting properties were studied using HER2+ SCOV3 xenograft models. PET/CT revealed significant accumulation of the radiotracer in the tumor within the first 2 h postinjection, providing a high tumor-to-background contrast (Figure 19B). Remarkably, there was an absence of a typical bone uptake, underscoring the exceptional *in vivo* stability of the compound 73.

In an alternative study, a radioiodine-based prosthetic group derived from 4-(5-methane-sulfonyl-[1,2,3,4]tetrazole-1-yl)-phenol (MSTP) was used for radiolabeling biomolecules. The direct radioiodination of MSTP using iodine-125 (^125^I; half-life, 60 days) resulted in the production of the ^125^I-labeled MSTP prosthetic group (Figure 19B) at high radiochemical yield (73%) and purity (>99%). Subsequently, ^125^I-labeled MSTP was used for the site-selective radiolabeling of biomolecules, including the GCQRPPR peptide and human serum albumin protein, at neutral pH and room temperature 74.

### Radiometal chelator-based POS

**Figure 20:**
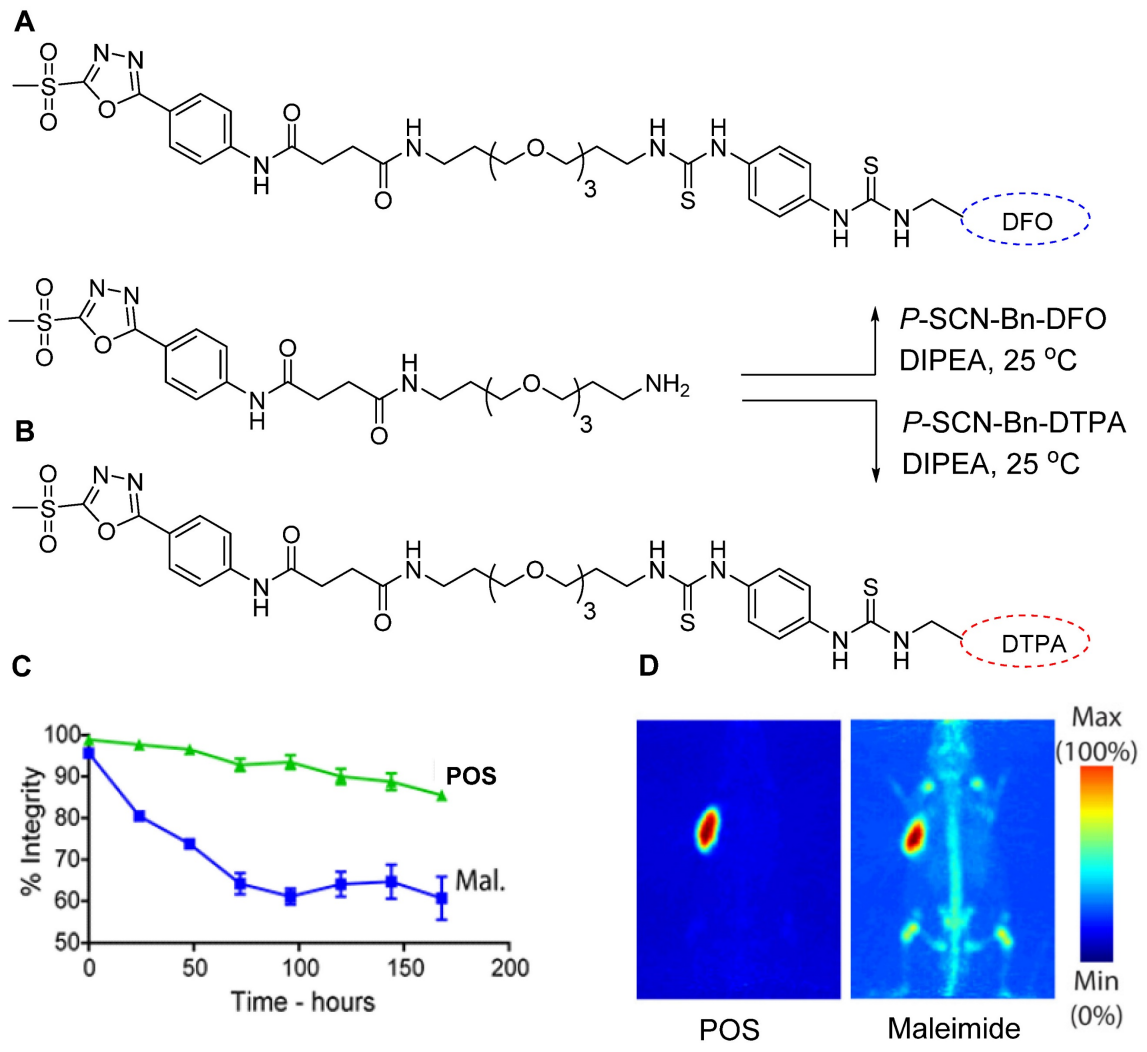
POS-based radiolabeling of biomolecules and *in vitro* and *in vivo* testing of resulting conjugates**.** (A) Synthesis of DFO-POS prosthetic group. (B) Synthesis of DTPA-POS prosthetic groups. (C) *In vitro* stability test of ^89^Zr-DFO-POS-trastuzumab compared with ^89^Zr-DFO-mal-trastuzumab under identical conditions. (D) PET images of A33 antigen-expressing SW1222 colorectal cancer xenograft bearing nude mice following the injection of ^89^Zr-DFO-POS-huA33 and ^89^Zr-DFO-mal-huA33 and images were acquired 120 h post injection. Adapted with permission from 75, copyright 2018, American Chemical Society.

Following the promising outcomes of ^18^F-FPOS-based radiolabeling of biomolecules, scientists have extended this technique to radiolabel biologically important molecules using radiometals. To facilitate this, radiometal-chelator-based POS prosthetic groups were synthesized, and commercially available compounds such as 5-phenyl-1,3,4-oxadiazole-2-thiol, and its derivatives, including 5-(4-aminophenyl)-1,3,4-oxadiazole-2-thiol, were explored. Among them, 5-(4-aminophenyl)-1,3,4-oxadiazole-2-thiol was selected and modified by incorporating a terminal amine-containing polyethylene glycol (PEG) group. The PEG group in addition to enhancing water solubility also provided additional space between the biomolecule and radioisotope chelator, which improved radiolabeling efficiency. The amine group in the modified compound was used to attach the bifunctional chelators DFO and DTPA (Figure 20A-B) for ^89^Zr and ^177^Lu radiolabeling, respectively 75. Both chelators were conjugated with a trastuzumab antibody via POS and thiol reactions. The final products, namely, ^89^Zr-DFO-POS-trastuzumab and ^177^Lu-DTPA-POS-trastuzumab, were obtained in high radiochemical yields and purities. *In vitro* stability studies revealed that both radiotracers exhibited superior stability for more than 7 days compared with that observed for the maleimide-based conjugate (Figure 20C). The huA33 antibody was also radiolabeled using either^89^Zr-DFO-Mal or ^89^Zr-DFO-POS before being intravenously injected into an A33 antigen-expressing SW1222 human colorectal cancer xenograft model for a comparative study. The PET images were acquired for several hours, demonstrating that the POS-based conjugate exhibited enhanced in* vivo* stability compared with that of its competitor (Figure 20D).

In 2020, Pieve *et al.* used the POS-thiol reaction for the ^18^F radiolabeling of antibodies using the [^18^F]AlF complex 76. They conjugated POS with NOTA and NODAGA chelators, resulting in the synthesis of NOTA-POS and NODAGA-POS prosthetic groups. Then, NOTA-POS was incorporated into the Z_EGFR:03115_-Cys antibody, which was subsequently radiolabeled using the [^18^F]AlF complex for a low radiochemical yield (11.0-12.7%) and high purity (>98%). The radiochemical yield of [^18^F]AlF-NODAGA-POS-Z_EGFR:03115_ was similarly low (4.3-8.1%). Moreover, the nature of the chelator significantly influenced the pharmacokinetics of both radiotracers, elevating the uptake in the liver, kidneys, and tumor. However, the biodistribution profiles of POS and the maleimide-based conjugates were almost identical up to 1 h postinjection. Despite the slow reaction kinetics, it is anticipated that POS-based radiolabeling strategies hold great potential in clinical settings.

### 2-cyanobenzothiazoles based radiolabeling

**Figure 21:**
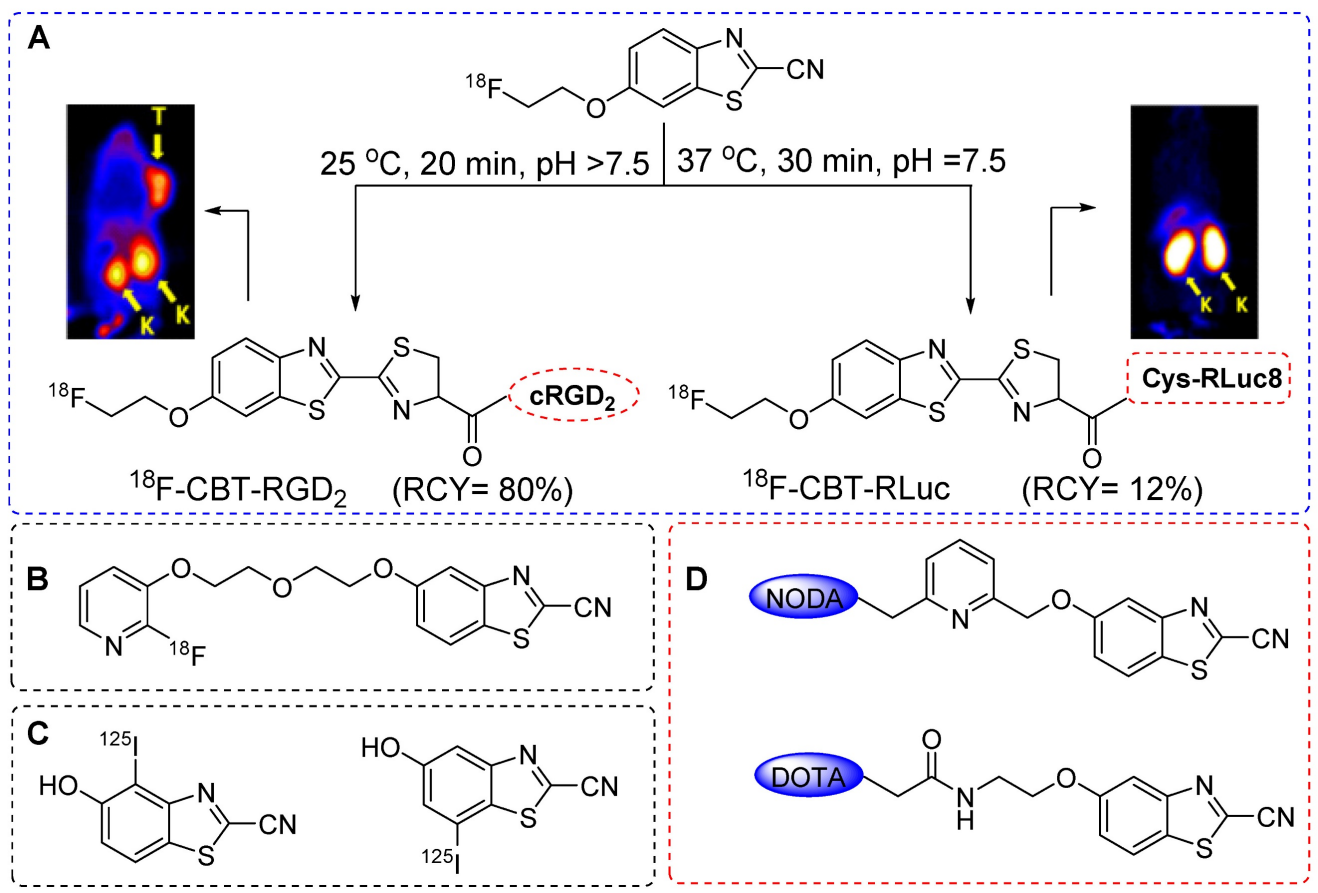
CBT-based radiolabeling of biomolecules and *in vivo* study. (A)^ 18^F-CBT based radiolabeling of dimeric c(RGD) peptide and PET images were acquired using U87MG tumor bearing (left side) 0.5 h post injection.^ 18^F-CBT was also used for the radiolabeling of Renilla lucifierase (RLuc8) protein and PET imaging were acquired using normal mouse (right side). T: tumors; K: kidneys. Adapted with permission from 81, copyright 2012, American Chemical Society. (B) Chemical structure of [^18^F]FPyPEGCBT. (C) Chemical structure of ^125^I-labeled CBT. (D) Chemical structure of DOTA and NODA based CBT prosthetic groups.

In Rao *et al.* reported for the first time a thiol-based condensation reaction between 2-cyanobenzothiazole (CBT) and an N-terminal cysteine residue to form luciferin linkage. This reaction occurred in PBS solvent under mild pH conditions and room temperature 77, 78. The reaction exhibited moderate kinetics with a second-order reaction rate of approximately 9.2 M^-1^s^-1^, which surpassed that of several well-defined click reactions reported previously 79,80. This reaction demonstrated site selectivity and specificity, which was further confirmed by labeling the N-terminal cysteine-containing proteins expressed on cell surfaces. As the reaction occurs exclusively at the N-terminal cysteine residue, its true strength lies in site selectivity but requires prior incorporation of the N-terminal cysteine in peptides and proteins. Chin *et al*. used this reaction to perform nuclear imaging and reported the ^18^F radiolabeling of N-terminal cysteine-containing proteins and peptides. The tosylate precursor was synthesized using commercially available 6-methoxy-CBT. However, the ^18^F radiolabeling attempts using [^18^F]KF-K_2.2.2_, tetrabutylammonium bicarbonate or Cs_2_CO_3_ were unsuccessful because of the hydrolysis of the cyano group on CBT. Nevertheless, successful radiolabeling was achieved using the 18-Crown-6/ K_2_CO_3_ complex at 90 °C for a moderate yield (20%) of ^18^F-CBT at high radiochemical purity. Furthermore, ^18^F-CBT was used to radiolabel the dimeric c(RGD) peptide with a high radiochemical yield (92% HPLC-based, 80% decay corrected) and purity (>98%) under physiological conditions. The PET imaging data from U87MG tumor-bearing mice showed high tumor targeting efficiency and high tumor-to-background ratio (Figure 21A). Elevated radioactivity signals were observed in the liver and kidneys owing to the increased lipophilicity of the radiolabeled compound resulting from the addition of the^18^F-CBT moiety. The efficacy of the prosthetic group was also determined using Renilla luciferase (RLuc8) protein after installing an N-terminal cysteine residue, which provided a moderate radiochemical yield (12%). PET images were acquired in normal mice, and the biodistribution profile of the radiolabeled protein was similar to that of ^124^I-labeled RLuc8 (Figure 21A) 81.

The ^18^F-CBT prosthetic group was synthesized using an aliphatic nucleophilic substitution reaction. Subsequently, new nucleophilic radiofluorination reactions were explored using heteroaromatic substituents, particularly those with a pyridine structure. ^18^F-CBT was more efficiently synthesized using nucleophilic heteroaromatic substitution than with aliphatic nucleophilic substitution. A new prosthetic group, [^18^F]FPyPEGCBT (Figure 21B) was synthesized using a trimethylammonium triflate precursor 82. The optimized radiolabeling conditions resulted in a high radiochemical yield (>45%) and purity (>98%). This prosthetic group was used to radiolabel the N-terminal cysteine-bearing c(RGD) peptide. Another study by Mushtaq *et al.* reported a ^125^I-labeled CBT for the radiolabeling of biomolecules 83. The direct radioiodination of CBT provided ^125^I-labeled CBT at high radiochemical yield (92%) and purity (>99%) within 10 min when a chloramine-T oxidizing agent was used (Figure 21C). The prosthetic group was tested on an N-terminal cysteine-containing c(RGD) peptide; however, the radiolabeled peptide was not subjected to further *in vivo* studies. Using the same theory, Seimbille *et al.* synthesized DOTA and NODAGA chelator-based CBT prosthetic groups (Figure 21D) for the efficient radiolabeling of biomolecules using ^68^Ga 84. Both chelators were tested for the radiolabeling of small peptides. Overall, the CBT-based radiolabeling of N-terminal cysteine-containing biomolecules represents an efficient, site-selective, and site-specific strategy with a high radiolabeling yield. However, the increase in the lipophilicity of molecules led to abnormal uptake in abdominal areas. Moreover, incorporating N-terminal cysteine into bioactive molecules before the radiolabeling reaction poses additional challenge in clinical settings.

## Thiol-ene click reaction based radiolabeling

**Figure 22:**
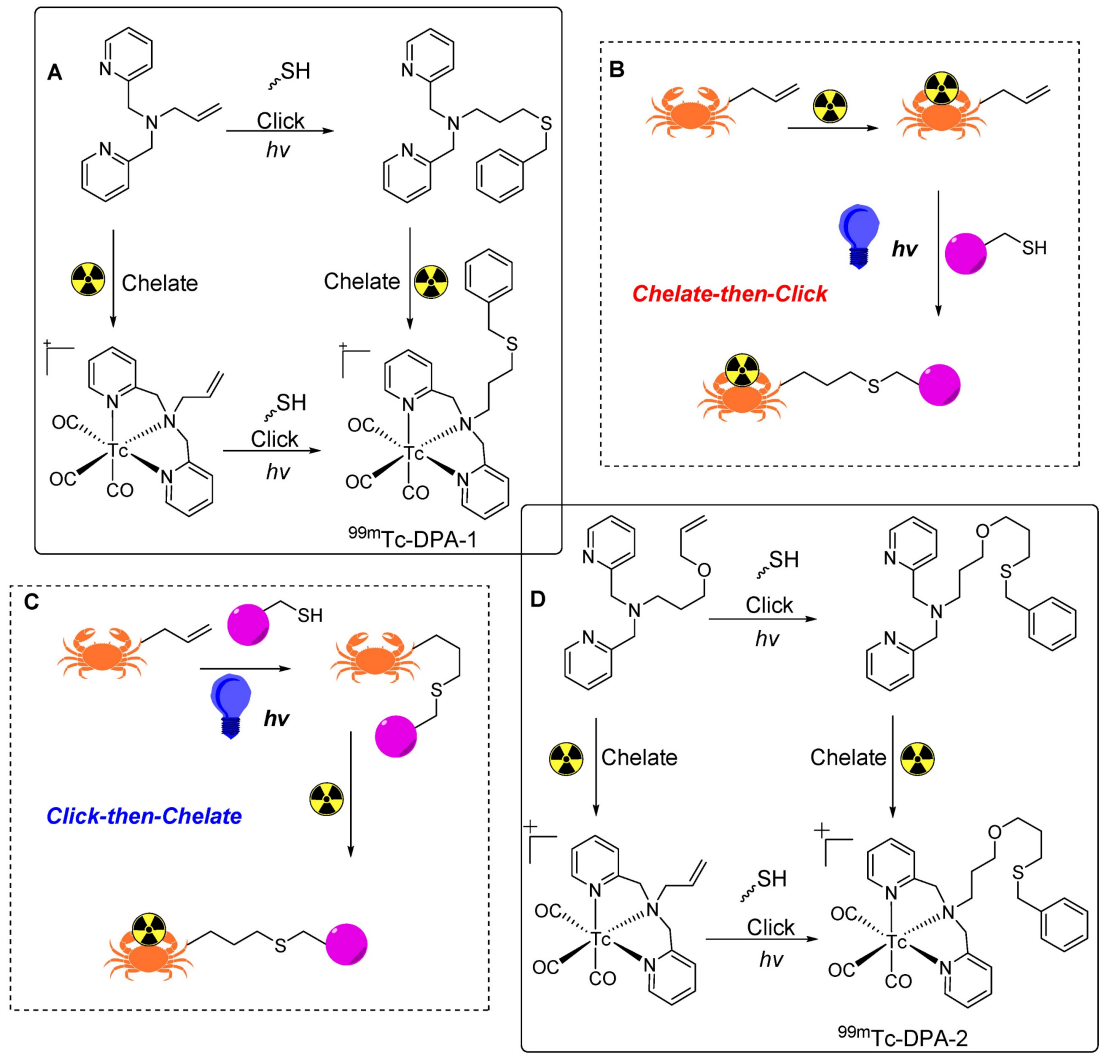
Thiol-ene click reaction based radiolabeling of biomolecules. (A) Synthesis of ^99m^Tc-labeled DPA-1 using click-then-chelate or chelate-then-click radiolabeling strategy. (B) Schematic illustration of chelate-then-click radiolabeling strategy. (C) Schematic illustration of click-then-chelate radiolabeling strategy. (D) Synthesis of ^99m^Tc-labeled DPA-2 using click-then-chelate or chelate-then-click radiolabeling strategy.

In the early 1900s, efficient and site-selective reactions were reported between free thiol and carbon-to-carbon double bonds or “enes”. These thiol-ene reactions exhibit the key attributes of a reliable click reaction. The photo induced thiol-ene reaction, characterized by ease of implementation, rapid kinetics, high yield, stable product formation, and photo initiation control, demonstrates potential in general synthetic chemistry and radiopharmacy 85. Initially used in the synthesis of macrocyclic compounds 86, sugars 87, peptides 88, dendrimers, and polymers 89, thiol-ene reactions have recently been used for the synthesis of ^99m^Tc based radiotracers. To achieve this, 2,2′-dipicolylamine (DPA) based BFCs were synthesized to link the central amine of DPA with the terminal alkene via a propyl allyl ether or allyl linker. The ^99m^Tc radiolabeled tracers were synthesized using either a click-then-chelate or a chelate-then-click strategy (Figure 22B,C). Benzyl mercaptan was used as a model thiol compound for photo-initiated thiol-ene conjugation reactions 90.

In the initial click-then-chelate approach, 3-(benzylthio)-N,N-bis(pyridin-2-ylmethyl)propan-1-amine (DPA-1) was synthesized by radiolabeling with fac-[^99m^Tc(OH_2_)_3_(CO)_3_]^+^ at 70 °C for 30 min, resulting in the production of ^99m^Tc-DPA-1 at a notable radiochemical yield of 89% (Figure 22A). The parallel synthesis of 3-(benzylthio)-N,N-bis(pyridin-2-ylmethyl)propan-1-amine (DPA-2) was performed using the same radiolabeling procedure for a superior 98% radiochemical yield (Figure 22D). In the chelate-then-click approach, N,N-bis(pyridin-2-ylmethyl)prop-2-en-1-amine was radiolabeled under the same conditions, and the corresponding radiolabeled complex was obtained at a 99% radiochemical yield. However, the use of 3-(allyloxy)-N,N-bis(pyridin-2-ylmethyl)propan-1-amine resulted in a 94% radiochemical yield. Subsequently, the thiol-ene reactions for the synthesis of ^99m^Tc-DPA-1 and ^99m^Tc-DPA-2 provided radiochemical yields of 64-88% and 52%, respectively. Thus, the chelate-then-click approach was suitable for temperature-sensitive molecules, although high thiol concentrations were necessary to achieve a high radiochemical yield. Further studies are needed to optimize thiol-ene reactions in terms of radiopharmacy.

## Thiol specific strained-release reaction

**Figure 23:**
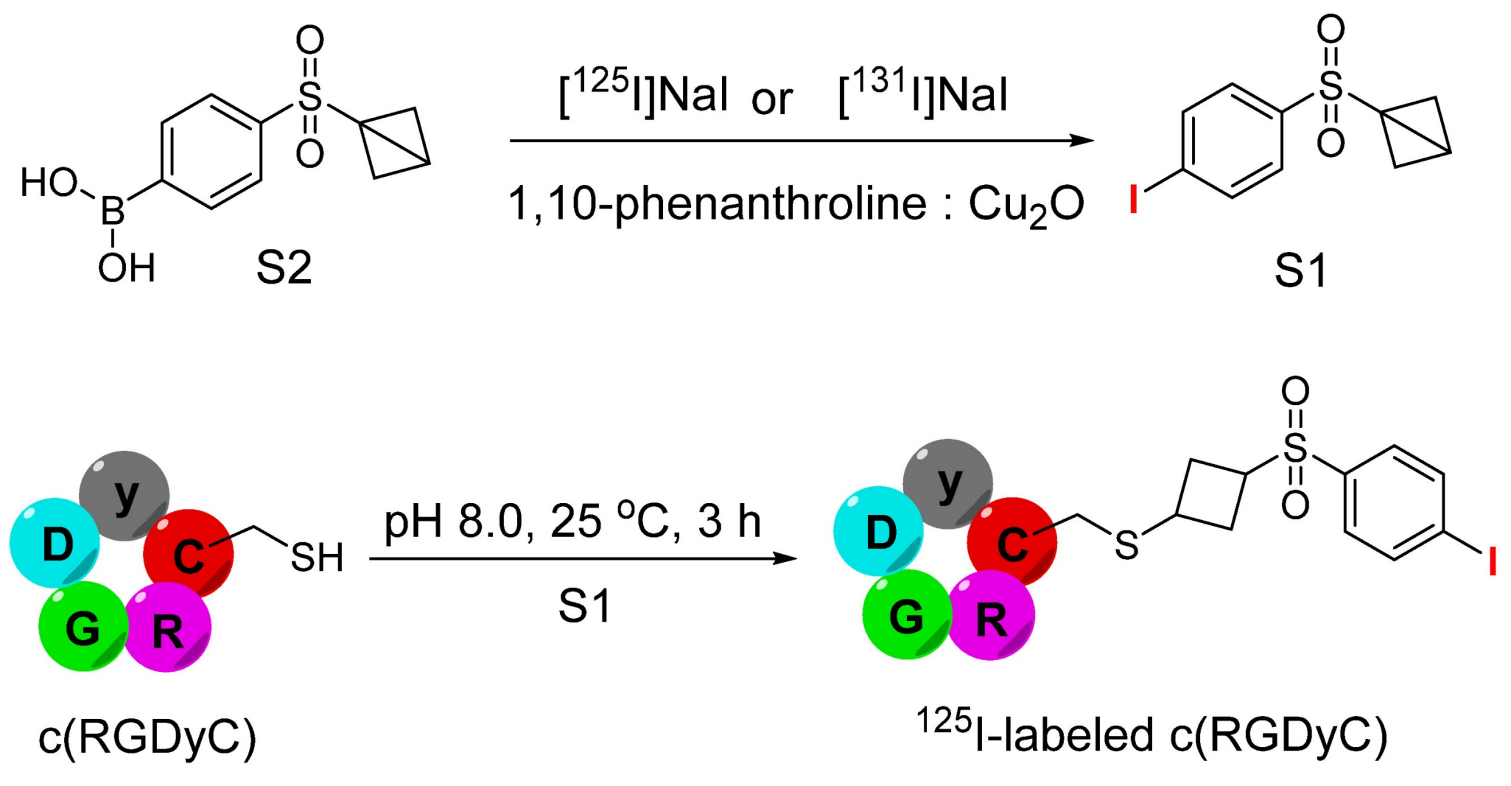
Schematic illustration of synthesis of 1-((4- [^131^I] iodophenyl)sulfonyl)bicyclo[1.1.0] butane or 1-((4-[^125^I]iodophenyl)sulfonyl)bicyclo[1.1.0]butane (S1) utilizing (4-(bicyclo[1.1.0]butan-1-ylsulfonyl)phenyl)-boronic acid (S2). The next step is demonstrating the synthesis of ^125^I-labeled c(RGDyC) peptide using ^125^I-labled S1 under basic conditions.

The strained bonds have long been recognized for their unique applications in organic syntheses. These molecules store significant potential energy and are suitable for versatile applications in biology, materials science, chemistry, and bioorthogonal chemistry 91. Notably, these molecules exhibit remarkable chemoselectivity for thiols and excellent stability under harsh reaction conditions 92. A new type of hetero-bifunctional agent was developed by leveraging these scaffolds for the radioiodination of thiol-containing peptides. Specifically, 1-((4- [^131^I]iodophenyl)sulfonyl)bicyclo[1.1.0]butane or 1-((4 [^125^I]iodophenyl)sulfonyl)bicyclo[1.1.0] butane (S1) was synthesized using (4-(bicyclo[1.1.0]butan-1-ylsulfonyl)phenyl)-boronic acid (S2) for radioisotope-based therapy and SPECT imaging, respectively (Figure 23). The radiolabeling process was catalyzed by Cu_2_O and yielded the desired product within 1 h at room temperature. Strain-release reactions with thiols were performed in phosphate buffer (pH = 8.0), and K_2_CO_3_ was used to ensure an alkaline pH, which facilitates the deprotonation of cysteine thiol in the peptide. Under optimized conditions, various peptides, including N-acetylcysteine, L-cysteine, L-cysteine methyl ester hydrochloride, CAQK, and c(RGDyC) were radiolabeled with a high radiochemical yield (>95%) and purity (>99%). The ^125^I-labeled c(RGDyC) was subjected to further *in vivo* and *in vitro* studies. The radioiodinated compound demonstrated high *in vitro* and *in vivo* stability and elevated uptake in the tumors of U87MG tumor-bearing mice 93. Despite these favorable results, the prosthetic group was not tested for the radiolabeling of macromolecules such as proteins and antibodies.

## Conclusion and future perspectives

Thus, this review presents our findings of what we perceive to be the most important and highly innovative thiol-based radiolabeling strategies. This comprehensive review primarily focuses on thiol-based prosthetic groups by examining their direct applications, analyzing radiolabeling outcomes, and considering their potential impact on the pharmacokinetics and binding affinity of radiolabeled biomolecules (summarized in Table 2). We acknowledge that the scientific data elaborated here primarily stems from preclinical studies. Additionally, some of the studies discussed here that feature thiol-based prosthetic groups do not use them directly but rather as intermediates to support or supplement other click reactions such as in the IEDDA or aldehyde and 1,2-diamine conjugation reactions; this aspect has not been explicitly addressed in this review. Similarly, we have intentionally omitted an in-depth discussion of reactions that use catalysts for the direct modification of thiol groups with radioisotopes such as ^18^F or ^11^C. These deliberate omissions highlight both the strengths and limitations of our study aim, warranting further studies with nuanced consideration to comprehensively understand the broad landscape of thiol-based radiolabeling strategies.

The intricate development of targeted nuclear imaging and radiotherapeutic agents necessitates a meticulous approach involving the precise incorporation of radioisotopes into biomolecules of interest with chemo- and site-specificity. The prosthetic group approach, particularly the modification of cysteine (Cys) residues, is the prevalently used strategy for radiolabeling thiol-containing biomolecules. Although significant progress has been achieved in radiolabeling using various prosthetic groups based on maleimide, vinylsulfone, aryl sulfone, CBT, and thiol-ene conjugation reactions, critical hurdles remain. These limitations include *in vivo* stability issues, reactivity towards non-thiol functional groups, sluggish reaction kinetics, and the need for excess substrate to achieve a sufficient radiolabeling yield. These challenges hinder the use of these prosthetic groups in preclinical and clinical settings.

Further developments leading to the use of thiol-based reactions in clinical nuclear medicine depend on overcoming the current limitations and fostering innovative applications. Efforts should be directed towards refining prosthetic groups, exploring new synthesis routes, and assessing their compatibility with other bioconjugation methods. The inherent potential of thiol-based reactions, especially those with short-lived radioisotopes such as ^18^F, ^11^C, and ^68^Ga, needs to be thoroughly investigated and compared through detailed studies.

As this field of application evolves, enhanced efficacy can be attained through the combination of thiol-based prosthetic groups and other click reactions. The continuous expansion of thiol-based reactions into clinical trials is not only desirable but also crucial for establishing their true impact. Ease of synthesis, site-specific bioconjugation, high stability, automation, and probe purification have emerged as key areas in which thiol-based reactions can revolutionize clinical nuclear medicine and offer novel and impactful solutions.

## Figures and Tables

**Table 1:** Comprehensive overview of maleimide-based prosthetic groups for radiolabeling: applications, strengths, and limitations.

S. No.	Prosthetic group	Applications	Strengths	Limitations	Ref.
1	[^18^F]FPPD	-	-	Low radiochemical yield and multistep synthesis.	21
2	[^18^F]DDPFB	Utilized for the^ 18^F radiolabeling of Fab derived from IgG.	Thiol specific radiolabeling.	Low radiochemical yield and multistep synthesis.	21
3	[^18^F]FBABM	Utilized for^ 18^F radiolabeling of GSH peptide and ODNs.	The application of the reaction between benzaldehyde and the aminooxy functional group resulted in a higher radiolabeling yield.	The process involved multistep synthesis and a complex radiolabeling procedure.	22
4	[^18^F]FBAM	Utilized for^ 18^F radiolabeling of GSH peptide and human low-density lipoprotein (LDL).	Improved lipophilicity was observed compared to [^18^F]FBABM.	The process involved multistep synthesis and a complex radiolabeling procedure.	23
5	[^18^F]FBOM	Utilized for^ 18^F radiolabeling of GSH peptide and human low-density lipoprotein (LDL).	Enhanced hydrophilicity was observed.	The process involved multistep synthesis, resulting in poor radiochemical yield.	24
6	[^18^F]FPyME	Utilized for^ 18^F radiolabeling of (*N*-Ac)-KAAAAC peptide.	Improved radiochemical yield and stability were achieved.	The process involved multistep synthesis, resulting in poor radiochemical yield.	25
7	[^18^F]FBEM	Utilized for^ 18^F radiolabeling of monomeric and dimeric c(RGD) peptides, GLP-1 analogue (EM3106B), , Cys^0^- exendin-4, and [Cys^40^]- exendin-4.	No impact on tumor binding affinity was observed, while enhanced *in vitro* and *in vivo* stability and high PET image quality were achieved.	The process involved multistep synthesis, resulting in poor radiochemical yield.	26, 29-31
8	[^18^F]FDG-MHO	Utilized for^ 18^F radiolabeling of GSH peptide and annexin V.	High *in vitro* and *in vivo* stability were observed alongside a high radiochemical yield.	The process involves multistep synthesis, resulting in a final product that is a mixture of isomers.	32
9	DOTA-malimide	Utilized for^ 90^Y and ^177^Lu-based radiolabeling of GSH peptide and 12 mer L-RNA.	High radiochemical yield was achieved with a straightforward synthesis.	Poor *in vivo* stability was observed after 48 hours.	36
10	DOTA- malimide	Utilized for^ 68^Ga-based radiolabeling of c(RGD) peptide, BSA, and folic acid.	High radiochemical yield was achieved with a straightforward synthesis.	Poor *in vivo* stability.	37
11	DFO- malimide	Utilized for^ 89^Zr-based radiolabeling of trastuzumab antibody.	High radiochemical yield was achieved with a straightforward synthesis.	Poor *in vivo* stability was observed after 48 hours.	43
12	H_4_neunox-maleimide	Utilized for^ 89^Zr-based radiolabeling of trastuzumab antibody.	High radiochemical yield was achieved with a straightforward synthesis.	Compared to DFO, the process resulted in low radiochemical yield and poor *in vivo* stability.	44
13	NOTA- maleimide	Utilized for^ 18^F-based radiolabeling of [Cys^40^]-exendin-4, Z_HER3:8698_-Cys, and Cys-annexin V protein.	High radiochemical yield was achieved with a straightforward synthesis.	The prosthetic group is unsuitable for the radiolabeling and *in vivo* study of macromolecules due to short half-life of ^18^F.	45, 47, 48
14	[^18^F]FPenM and [^18^F]FNEM	Utilized for^ 18^F-based radiolabeling of [Cys^40^]-exendin-4	The radiolabeled compound exhibited superior pharmacokinetics.	The multistep synthesis entails a complex procedure and yields poor radiochemical results.	49, 50

**Table 2:** Comprehensive overview of prosthetic groups used for radiolabeling thiol-containing biomolecules: applications, strengths, and limitations.

S. No.	Prosthetic group/ radiolabeling conditions	Applications	Strengths	Limitations	Ref.
1	**Maleimide** Reaction conditions: pH: 7.0 to 7.5 Media: 10% DMF in aqueous buffer Temperature: 25 °C Time: 10 to 90 min Additive: 5-10 eq TCEP	Used for the ^18^F, ^68^Ga, ^90^Y, ^177^Lu, or ^89^Zr-based radiolabeling of peptides, proteins, and antibodies.	Exhibiting fast reaction kinetics, high specificity to the cysteine moiety, and the ability to react under physiological conditions, it can be attached to various ^18^F precursors and radiometal chelators.	***For ^18^F radiolabeling*** The synthesis is multistep and complex; however* in vivo* stability supports imaging study. ***For ^68^Ga radiolabeling*** The synthesis is straightforward and facile, with *in vivo* stability supporting imaging studies. However, it cannot be employed for the imaging of macromolecules due to its short half-life. ***For ^89^Zr, ^90^Y, and ^177^Lu radiolabeling*** The synthesis is easy and simple; however, it exhibits poor *in vivo* stability after 48 h.	21-50
2	**Vinyl sulfone** Reaction conditions: pH: 8.0 to 8.5 Media: 10% DMF in aqueous buffer Temperature: 25 °C Time: 5 to 30 min Additive: 5-10 eq TECP	Used for the ^18^F, ^64^Cu, and ^111^In-based radiolabeling of peptides, proteins and antibodies.	The prosthetic groups demonstrate a simple and easy synthesis, offering moderate to high radiochemical yield. They react with thiol under physiological conditions and can be attached to various ^18^F precursors and radiometal chelators, ensuring high *in vivo* and *in vitro* stability of both the prosthetic groups and the final radiolabeled product.	Exhibiting slower reaction kinetics compared to the maleimide-thiol reaction, it is not specific to cysteine only and can react with lysine under certain conditions.	54-63
3	**Phenyl vinyl sulfone** Reaction conditions: pH: 8.5 Media: 50% Methanol in borate buffer Temperature: 30-35 °C Time: 30-40 min Additive: 2 mM TECP	Used for the ^18^F-based radiolabeling of peptides.	The prosthetic groups demonstrate a simple and easy synthesis, offering moderate to high radiochemical yield. They react with thiol under physiological conditions and can be attached to various ^18^F precursors.	Exhibiting slower reaction kinetics compared to the maleimide-thiol reaction, it is not specific to cysteine only and can react with lysine under certain conditions.	64-65
4	**Perfluoroarylation** Reaction conditions: pH: 7.0 to 7.5 Media: DMF Temperature: 25°C Time: 45 min Additive: 2 mM TCEP	Used only for the ^18^F-based radiolabeling of peptides.	Featuring a simple and easy synthesis, it exhibits high *in vivo* stability, and allows for simultaneous radiolabeling and dimerization of peptides.	The reaction has slow kinetics and overall low radiochemical yield. It is only suitable for the radiolabeling of peptides.	69
5	**Aryl sulfone** Reaction conditions: pH: 8.5 Media: 10-50% DMSO in borate buffer Temperature: 30-35 °C Time: 30-120 min Additive: 10 eq TECP	Used for the ^18^F, ^125^I, and ^89^Zr- based radiolabeling of peptides, proteins, and antibodies.	The prosthetic groups undergo a simple and easy synthesis, offering moderate to high yields. They react with thiols under physiological conditions and can be attached various ^18^F precursors and radiometal chelators, ensuring high *in vivo and in vitro* stability for both the prosthetic groups and the final radiolabeled product.	Exhibiting slower reaction kinetics compared to the maleimide-thiol reaction, it is not specific to cysteine only and can react with lysine under certain conditions.	73-76
6	**2-Cyanobenzothiazoles** Reaction conditions: pH: 7.0 to 7.5 Media: 10% DMF in aqueous buffer Temperature: 37°C Time: 30 min Additive: 2 mM TCEP	Used for the ^18^F, ^125^I, and ^68^Ga- based radiolabeling of peptides, and proteins.	The prosthetic groups undergo a simple and easy synthesis, yielding radiochemical results ranging from moderate to high. They react with N-terminal cysteine under physiological conditions, can be attached to various ^18^F precursors and radiometal chelators, and demonstrate high *in vivo* and *in vitro* stability for both the prosthetic groups and the final radiolabeled product. Notably, they are highly specific towards N-terminal cysteine.	Special modification is required to incorporate N-terminal cysteine into the peptide and protein to be radiolabeled. Bulky conjugation increases the lipophilicity of the radiolabeled products, impacting their binding efficiency and pharmacokinetics.	81-84
7	**Thiol-ene** Reaction conditions: pH: > 7.0 Media: MeOH Temperature: 25°C Time: 60 min Special conditions: Dimethylolpropionic acid and UV irradiation at 366 nm	Used for the ^99m^Tc- based radiolabeling of small molecules.	The prosthetic group is highly stable under harsh reaction conditions, allowing for both click-then-chelate and chelate-then-click strategies, resulting in moderate to high radiochemical yield.	Exhibiting slow reaction kinetics, there is currently no available radiolabeling data for macromolecules, and data on *in vitro* and *in vivo* stability are not yet available.	90
8	**Strained release reaction** Reaction conditions: pH: 8.0 Media: 10%DMF in aqueous buffer Temperature: 60°C Time: 30 to 60 min Special conditions: 0.2 M potassium carbonate	Used for the radioiodine-based radiolabeling of small molecules.	A high radiochemical yield is achieved for peptide radiolabeling, along with high *in vivo* and *in vitro* stability.	The synthesis involves a multi-step complex process and has only been attempted for radioiodine-based radiolabeling of peptides. The reaction kinetics is slow, necessitating high temperatures to complete the reaction with thiol. However, no data are available for protein and antibody radiolabeling.	93

## Abbreviations

NHS ester: *N-*Hydroxysuccinimide esters
ADCs: Antibody-drug conjugates
FDA: Food and Drug Administration
HER2: Human epidermal growth factor receptor 2
Lys: Lysine
Cys: Cysteine
[^18^F]FPPD: 1-(4-[^18^F]Fluorophenyl)-pyrrole-2,5-dione
[^18^F]DDPFB: *N*-[3-(2,5-dioxo-2,5-dihydropyrrol-1-yl)-phenyl]-4-[^18^F]fluorobenzamide
MBS: m-Maleimidobenzoyl N-hydroxysuccinimide ester
LDL: Human low-density lipoprotein
[^18^F]FBABM: (*N*-[4-[(4-[^18^F]fluorobenzylidene)aminooxy]butyl]maleimide
[^18^F]FBAM: N-[6-(4-[^18^F]Fluoro-benzylidene)aminooxyhexyl]maleimide
[^18^F]FBOM: 4-[^18^F]Fluorobenzaldehyde-O-(2-{2-[2-(pyrrol-2,5-dione-1-yl)ethoxy]-ethoxy}-ethyl)oxime
[^18^F]FPyME: 1-[3-(2-[^18^F]Fluoropyridin-3-yloxy)propyl]pyrrole-2,5-dione
[^18^F]FBEM: N-[2-(4-^18^F-fluorobenzamido)ethyl]maleimide
GLP-1: Glucagon-like peptide-1
INS: Insulinoma
[^18^F]FBAPM: 4-[^18^F]Fluorobenzylamidopropionyl maleimide
[^18^F]FDG: [^18^F]Fluoro-2-deoxy-D-glucose
BFCs: Bifunctional chelators
DOTA: (1,4,7,10-Tetraazacyclododecane-1,4,7,10-tetrayl)tetraacetic acid
Df or DFO: Desferrioxamine B
^68^Ga: Gallium-68
^111^In: Indium-111
IEDDA: Inverse electron demand Diels-Alder
HER3: Human epidermal growth factor receptor 3
MSBT: 2-(Methanesulfonyl)benzothiazole
POS: Phenyloxadiazole methylsulfone
BBN: Bombesin
PET/CT: Positron emission tomography and computed tomography
MSTP: 4-(5-Methane-sulfonyl-[1,2,3,4]tetrazole-1-yl)-phenol
PEG: Polyethylene glycol
DTPA: Pentetic acid or diethylenetriaminepentaacetic acid
CBT: 2-Cyanobenzothiazole
DPA: 2,2′-Dipicolylamine
TCEP: Tris(2-carboxyethyl)phosphine hydrochloride
